# The upstream 5′ splice site remains associated to the transcription machinery during intron synthesis

**DOI:** 10.1038/s41467-021-24774-6

**Published:** 2021-07-27

**Authors:** Yodfat Leader, Galit Lev Maor, Matan Sorek, Ronna Shayevitch, Maram Hussein, Ofir Hameiri, Luna Tammer, Jonathan Zonszain, Ifat Keydar, Dror Hollander, Eran Meshorer, Gil Ast

**Affiliations:** 1 Department of Human Molecular Genetics and Biochemistry, Sackler Faculty of Medicine, Tel-Aviv University, Ramat Aviv, Israel; 2 Department of Genetics, The Institute of Life Sciences, and The Edmond and Lily Center for Brain Sciences (ELSC), The Hebrew University of Jerusalem, Edmond J. Safra Campus, Jerusalem, Israel

**Keywords:** Alternative splicing, RNA splicing

## Abstract

In the earliest step of spliceosome assembly, the two splice sites flanking an intron are brought into proximity by U1 snRNP and U2AF along with other proteins. The mechanism that facilitates this intron looping is poorly understood. Using a CRISPR interference-based approach to halt RNA polymerase II transcription in the middle of introns in human cells, we discovered that the nascent 5′ splice site base pairs with a U1 snRNA that is tethered to RNA polymerase II during intron synthesis. This association functionally corresponds with splicing outcome, involves bona fide 5′ splice sites and cryptic intronic sites, and occurs transcriptome-wide. Overall, our findings reveal that the upstream 5′ splice sites remain attached to the transcriptional machinery during intron synthesis and are thus brought into proximity of the 3′ splice sites; potentially mediating the rapid splicing of long introns.

## Abstract (web summary)

We know that most splicing reactions take place co-transcriptionally, but how the transcription machinery facilitate splicing of introns is unknown. Here the authors show that the 5′ splice site remains associated with the transcription machinery during intron synthesis through U1 snRNP, providing a basis for the rapid splicing reaction of introns.

## Introduction

Splicing is the mRNA maturation reaction where introns are removed from pre-mRNA and exons are ligated together^1,2^. The splicing machinery recognizes either exons or introns as the spliced unit, through mechanisms called exon definition and intron definition, respectively^3^. Splicing is carried out within the spliceosome, a multi-component complex composed of five nuclear ribonucleoprotein (snRNP) complexes—U1, U2, U4, U5, and U6—and many additional proteins^4,5^. The splicing reaction is governed by four main regulatory consensus sequences: the 5′ and the 3′ splice sites (5′SS and 3′SS, respectively), which are located at exon–intron boundaries, the polypyrimidine tract (PPT), and the branch site sequence. The PPT and the branch site are located upstream of intronic 3′ ends^6^. The first step in spliceosome assembly is the formation of the commitment complex. In this complex, the U1 snRNP binds the 5′SS via base pairing between U1 snRNA and the 5′SS, and the 3′SS and the PPT are associated with a heterodimer of U2AF1 (U2AF35) and U2AF2 (U2AF65)^7,8^. Both splice sites are thus defined at this early stage of the reaction. The commitment complex then advances into the pre-spliceosome (complexes A and B), which transitions to other complexes that catalyze intron removal and exon ligation in two steps (complex C)^6^.

Recent studies indicate that most pre-mRNAs undergo splicing while being transcribed by RNA polymerase II (pol II)^9–12^, although there are exceptions^13^. This is termed co-transcriptional splicing. The C-terminal domain (CTD) of pol II is necessary for activation of transcription and for efficient pre-mRNA processing^14,15^. U1 snRNP and U2AF2 associate with the pol II CTD, and these interactions have functional effects on splicing^16,17^. The 5′SS and the PPT interact with U1 snRNP and U2AF2, respectively, immediately after emerging from within pol II^18,19^.

A study of splicing kinetics revealed that a large fraction of intron removal is complete within seconds^20,21^ to several minutes in living cells^13,22–24^. During vertebrate evolution introns lengthened by thousands of nucleotides, whereas the average exon length has remained about 150 nucleotides^25,26^. Intron lengthening was accompanied by only a minor compromise in splicing efficiency^25,27,28^: In mammalian cells, short and long introns are generally spliced rapidly irrespective of length^23,28^. This implies that the formation of the commitment complex occurs almost instantly following the synthesis of the 3′SS. However, the mechanism that brings the two splice sites into proximity to facilitate co-transcriptional splicing is unknown.

To interrogate this mechanism, we developed a CRISPR interference-based assay that enables the analysis of factors located over particular DNA regions that are associated with particular regions of the pre-mRNA. Our findings suggest the following model for co-transcriptional splicing: The U1 snRNP associates with elongating pol II during transcription. Once the 5′SS is synthesized, the U1 snRNA base pairs with the 5′SS and remains tethered to pol II. The U1 snRNP, the 5′SS, and elongating pol II progress together along the intron to the downstream 3′SS. These interactions result in intron looping between the two splice sites and facilitate accurate and rapid splicing.

## Results

### During transcription of downstream introns, pol II associates with the pre-mRNA 5′SS

**Fig. 1:**
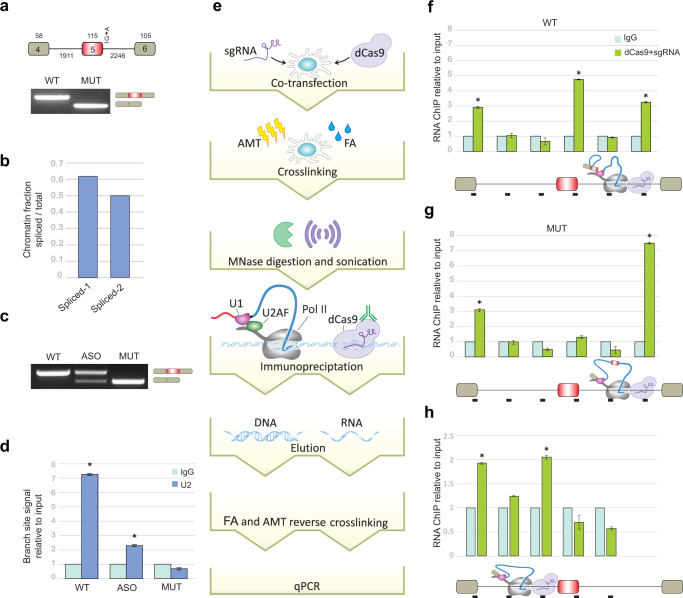
The 5′SS regions of pre-mRNAs are associated with pol II located in the middle of the downstream intron. **a** Upper panel: Diagram of *FRG1* minigene. 5′SS + 1 position mutation from G to A. Exon numbers and the exon and intron lengths are indicated. Lower panel: RT-PCR analysis of *FRG1* WT and MUT cells. Source data are provided as a Source Data file. **b** Amount of chromatin-associated RNA determined by qRT-PCR with exon–exon junction quantity divided by the sum of exon–exon and exon–intron junctions quantity^29^. One experiment was done. Spliced-1 denotes the exon 1–exon 2 junction and Spliced-2 denotes the exon 2–exon 3 junction. **c** Cells that express WT *FRG1* were treated with or without 750 nM of antisense oligonucleotide (ASO) complementary to the 5′SS region of intron 2 of the *FRG1* minigene. After 48 h, RNA was extracted, and the splicing pattern was examined by RT-PCR for ASO-treated cells, for WT and MUT cell lines. **d** RNA-ChIP analysis with anti-U2 snRNP antibody and IgG antibody as negative control were performed in WT, WT ASO-treated, and MUT cells. qRT-PCR was performed to quantify the amount of branch-site region from the first intron that was precipitated. *N* = 3 independent experiments. Error bars show mean values ± SD. Asterisk indicates for WT *P* = 0.006 and for ASO *P* = 0.005, two-tailed *t*-test. **e** Schematic overview of our CRISPR interference-based protocol. Cells are co-transfected with plasmids for expression of HA–dCas9 and sgRNA complementary to the desired location in a gene. After 48 h, cells are treated with FA and AMT, and nuclei are purified. Chromatin is digested with MNase and sonicated. Immunoprecipitation is performed with an anti-HA antibody, followed by RNA or DNA extraction, and real-time PCR analyses. **f**–**h** CRISPR interference-based experiments were performed with anti-HA antibody and IgG antibody as a negative control to evaluate the association of various transcript regions with pol II located **f** mid-intron 2 of WT *FRG1*, **g** mid-intron 2 of MUT *FRG1*, and **h** mid-intron 1 of WT *FRG1*. Mean RNA levels were measured. *N* = 3 independent experiments. Each bar corresponds to the amplified segment marked in the gene diagram below the graph. Error bars show mean values ± SEM. Asterisk indicates from left to right for **f** *P* = 0.002, 8 × 10^−^^4^, 0.001, for **g** *P* = 0.01, 5 × 10^−6^, for **h** *P* = 0.004, 0.02, two-tailed *t*-test.

To study co-transcriptional splicing when pol II is located at specific genomic locations, we developed a CRISPR interference-based approach. We first stably introduced a segment of the human *FRG1* gene containing three exons and two long introns into Flp-In-HEK293 cells; these cells are hereafter referred to as the wild-type (WT) cells. The same segment containing a point mutation at the 5′SS of the second intron was also introduced into Flp-In-HEK293 cells to construct a mutant (MUT) cell line. The mutation changes the splicing pattern of the middle *FRG1* exon from inclusion to skipping (Fig. 1a). In order to examine whether *FRG1* transcripts are co-transcriptionally spliced, the cells were fractionated^29^ (Supplementary Fig. 1a), and qRT-PCR on chromatin-associated RNA demonstrates that *FRG1* splicing is carried out co-transcriptionally (Fig. 1b). We next sought to determine whether the binding of U2 snRNP to *FRG1* transcripts is affected by the downstream 5′SS. Therefore, the 5′SS of the second intron was sequestered using an antisense oligonucleotide (ASO). We performed RNA-ChIP-qPCR using an anti-U2 snRNP antibody on extracts of WT and MUT cells and on extracts of WT cells treated with the ASO. When the U1 interaction with the splice site was disrupted by ASO treatment, exon 2 was skipped in about 30% of transcripts (Fig. 1c) and U2 snRNP binding to the upstream branch site sequence was decreased (Fig. 1d). These data demonstrate that this exon is selected via the previously described exon-definition mechanism^30^. We also examined U2AF2, the protein that recognizes the PPT, using RNA ChIP in WT and MUT cells. Mutating the 5′SS of intron 2 increased U2AF2 binding to the PPT of intron 1 (Supplementary Fig. 1b). This increased binding of U2AF2 to the PPT of the first intron likely reflects recognition of this site as a 3′SS although it is unused in splicing (a cryptic site). Thus, unlike the binding of U2 snRNP to the upstream branch site sequence, which is affected by U1 snRNP binding to the downstream 5′SS, the binding of U2AF2 to the upstream PPT is independent of the binding of U1 snRNP to the downstream 5′SS. Independent binding of U2AF2 to the PPT was also shown in an in vitro system^31^. These results indicate that the binding of U1 snRNP to the downstream 5′SS is important for U2 snRNP binding at the upstream branch site, resulting in the formation of the cross-exon complex.

To stall pol II in the middle of the *FRG1* intron 2, over 1 kb from upstream and downstream splice sites, we used two sgRNAs complementary to the middle of the intron to direct the catalytically inactive HA–dCas9^32^ to this genomic location. Binding of the HA–dCas9 halts transcription in the middle of the intron (Supplementary Fig. 1c). Both HA–dCas9 and pol II were located on the same DNA fragment as shown by western blot (Supplementary Fig. 1d). sgRNAs binding efficiency was confirmed by ChIP-qPCR (Supplementary Fig. 1e).

To study RNA–RNA interactions associated with pol II, cells transfected with plasmids for expression of sgRNAs and HA–dCas9 were crosslinked with formaldehyde (FA) and psoralen derivative 4′-aminomethyltrioxsalen (AMT). FA crosslinks proteins–protein, protein–DNA, and protein–RNA interactions, whereas AMT intercalates into RNA duplexes and, upon irradiation with 365 nm UV light, generates inter-strand adducts between juxtaposed pyrimidine bases to crosslink RNA–RNA interactions^33^ (Supplementary Fig. 2a). Under our crosslinking conditions, U1 snRNA was the most abundant snRNA found with elongating and pausing pol II (identified as the serine 2-phosphorylated (p-Ser2) and serine 5-phosphorylated (p-Ser5) forms of the pol II CTD, respectively^10,34^) (Supplementary Fig. 2b, c). Furthermore, using co-immunoprecipitation with both forms of pol II, U1 snRNP and U2AFs are found to associate with pol II in an RNA-independent manner (Supplementary Fig. 2d, e).

Following crosslinking, nuclear extracts were prepared and subjected to nuclease digestion, sonication, and immunoprecipitation with an antibody that binds the HA tag of dCas9 (Fig. 1e). Using this strategy, we were able to examine fragments of RNA and DNA of less than 500 bp that are associated with pol II in living cells (Supplementary Fig. 1f). When we targeted the middle of intron 2 with our CRISPR interference-based protocol, we detected specific interactions with 5′SSs of the first and second introns as well as the sgRNA target area and observed no interactions with other intronic regions (Fig. 1f). This demonstrates that the mechanism of co-transcriptional splicing is governed by the attachment of the 5′SS to pol II as it transcribes the downstream intron. Strikingly, in the MUT *FRG1* transcript, the 5′SS of the first exon was significantly enriched in the pol II precipitate, but the 5′SS of the skipped exon was not (Fig. 1g). The mutation at the 5′SS + 1 position is from purine to purine which does not affect AMT crosslinking outcome. Therefore, the results indicate that only the functional 5′SS is associated with pol II. The 5′SSs level in the WT cells was confirmed by quantification of the absolute amounts of each of the two exon–intron junctions in the pre-mRNA by qRT-PCR. The second 5′SS level was higher compared to the first 5′SS (Supplementary Fig. 1g). These findings provide the first experimental evidence that tethering of an upstream 5′SS to pol II as it transcribes the downstream intron is correlated with splicing outcome.

To examine the involvement of the PPT in the tethering of the 5′SS with pol II, we targeted two sgRNAs to the middle of intron 1 and repeated the CRISPR interference-based protocol. There is no upstream PPT in the middle of intron 1, and the downstream PPT is not yet transcribed. We detected 5′SS enrichment of the first intron, suggesting that the association of the 5′SS of intron 1 with pol II does not require the PPT (Fig. 1h). Also, knockdown of U2AF2 (~50%) had no effect on the binding of the 5′SS of intron 2 to pol II in the middle of intron 2 (Supplementary Fig. 3a, b). The binding of U1 snRNA to the 5′SS independent of the PPT and U2AFs was also shown in in vitro systems^35^.

**Fig. 2:**
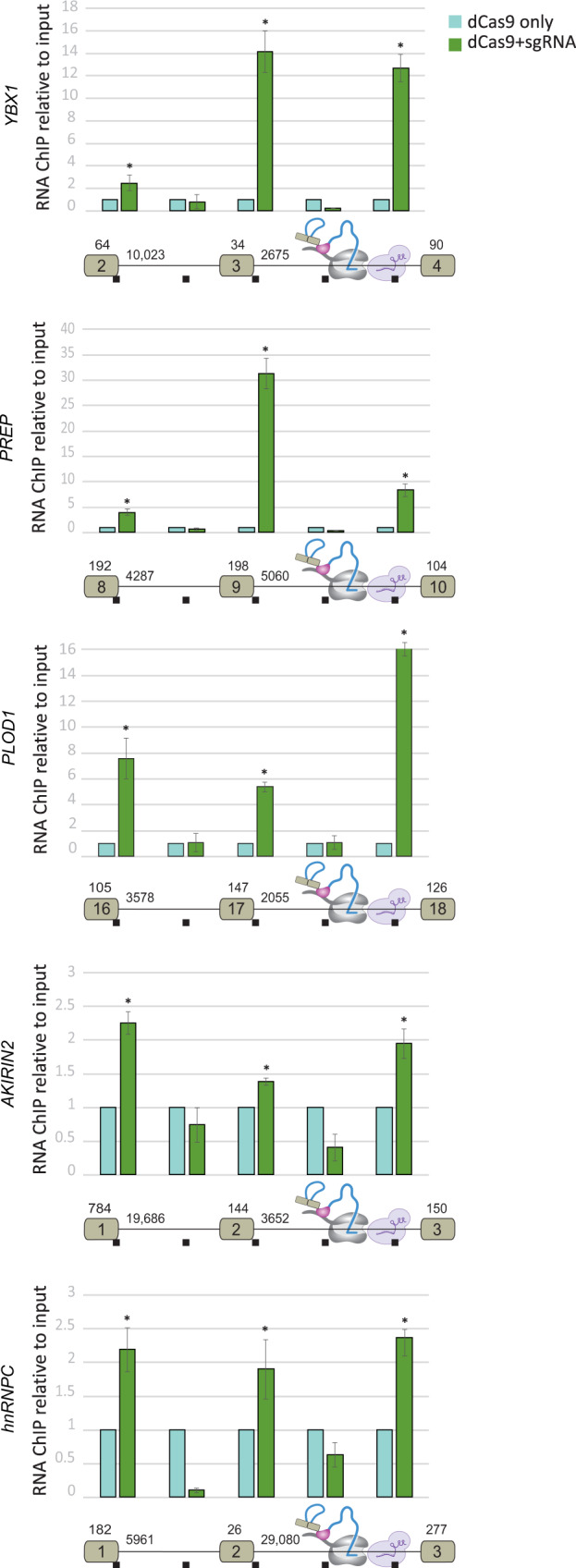
The 5′SSs of endogenous pre-mRNAs are associated with pol II located in the middle of the downstream intron. The CRISPR interference-based protocol was used to evaluate five endogenous genes (as shown in Fig. 1e). Exon numbers and intron and exon lengths are indicated. Mean RNA levels were measured. *N* = 3 independent experiments. Each bar corresponds to the amplified segment marked in the gene diagram below the graph. Error bars show mean values ± SEM. Asterisk indicates from left to right for *YBX1 P* = 0.05, 0.002, 8 × 10^−4^, for *PREP P* = 0.01, 5 × 10^−4^, 0.004, for *PLOD1 P* = 0.01, 3 × 10^−4^, 5 × 10^−4^, for *AKIRIN2 P* = 0.002, 0.002, 0.01, for *hnRNPC* one-tailed *t*-test *P* = 0.01, 0.03, 0.001, two-tailed *t*-test.

To extend these findings to other genes, we examined the association of an upstream 5′SS and intronic sequences with downstream intron sequences in five endogenous genes. These genes contain long introns, are highly expressed in HEK293 cells, and the relevant exons are constitutively spliced (Supplementary Fig. 4a). We used sgRNAs targeting the middle of the indicated introns (Fig. 2). sgRNAs binding was validated by immunoprecipitating dCas9 using ChIP-qPCR with an HA tag antibody (Supplementary Fig. 4b–f). We compared sgRNAs transfected to non-transfected cells and found enrichment of both 5′SS’s upstream exons and the sites of the sgRNAs, but no enrichment for intronic sequences (Fig. 2). Interestingly, the 5′SSs of the first and second introns were found to associate with pol II located in the middle of the downstream intron. The different proportions between the two 5′SSs in each gene might be due to the order and efficiency of the splicing reaction, and to the crosslinking affinity of AMT to each of the 5′SSs (see “Discussion” section for how multiple 5′SSs can be simultaneously associated with pol II). These findings support the generality of the association of the upstream 5′SSs to pol II as it transcribes downstream introns.

### The 5′SS is tethered to pol II through base pairing with U1 snRNA

**Fig. 3:**
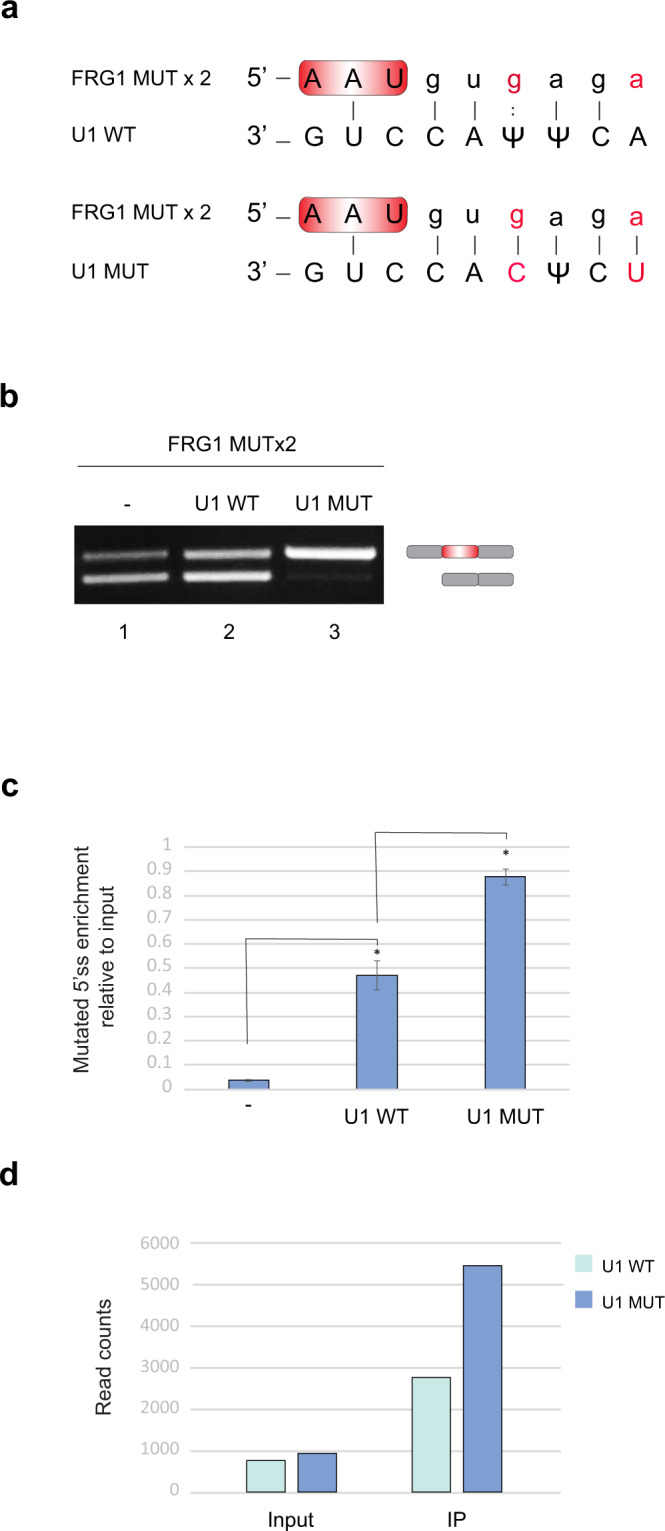
The U1 snRNA-5′SS-pol II interactionlocated in the middle of an intron is necessary for splicing. **a** Base pairing interaction between the mutant *FRG1* (A to G and T to A at positions +3 and +6 in the second 5′SS, termed *FRG1* MUTx2) and WT and MUT U1. A full line and colon indicate canonical and non-canonical base-pairing interactions, respectively. **b** Lane 1: RT-PCR analysis with specific primers to exons 1 and 3 of RNA extracted from cells transfected *FRG1* MUTx2 minigene. Lanes 2 and 3: RT-PCR analysis of RNA extracted from cells co-transfected with *FRG1* MUTx2 minigene and with either WT U1 snRNA or MUT U1, respectively. The splicing products were analyzed by gel electrophoresis. Source data are provided as a Source Data file. **c** The CRISPR interference protocol was performed to quantify the amount of the 5′SS of intron 2 of the *FRG1* MUTx2 located in the middle of intron 2 without or with co-transfection of WT or MUT U1 snRNA. Plotted are means of *n* = 3 independent experiments. Error bars show mean values ± SEM. Asterisk indicates from left to right *P* = 0.002, 0.004, two-tailed *t*-test. **d** CRISPR interference protocol was performed after co-transfection of *FRG1* MUTx2 and mutated U1 snRNA with compensatory mutations. The eluted RNA was sequenced and reads were mapped to U1 MUT and WT reference sequences. The bar plot shows the read counts in positions +3 to +6 of the U1 MUT and WT. χ^2^ test of independence was applied (*P* = 1.92e−18). One replicate was done.

To identify the mechanism by which the 5′SS is attached to pol II, we examined the base pairing of the upstream 5′SS with U1 snRNA, when the U1 snRNP attached to pol II is located in the middle of an intron. We used a genetic approach in which we generated mutations at the 5′SS of exon 2 in the *FRG1* minigene (A to G and T to A at positions +3 and +6, respectively), termed *FRG1* MUTx2, (Fig. 3a). The *FRG1* MUTx2 resulted in skipping of exon 2 in ~55% of mature mRNAs (Fig. 3b, lane 1). Next, we created mutations in U1 snRNA to complement the mutated 5′SS. In cells that express the *FRG1* MUTx2, co-transfection with the plasmids that express the WT or the MUT U1 snRNAs revealed that only the MUT U1 snRNA restored full exon 2 inclusion, whereas overexpression of WT U1 enhanced the amount of the two isoforms without changing the ratio between them (Fig. 3b lane 2 and 3). This was expected as overexpression of U1 snRNA increases the amount of U1 snRNP^36^, which further stabilizes spliceosome assembly and splicing outcome^37^. These results indicate that complementary base pairing between the mutated 5′SS and mutated U1 snRNA is necessary for the inclusion of exon 2.

Next, we examined the presence of base pairing between the mutated 5′SS and the mutated U1 snRNA associated with pol II located in the middle of the downstream intron using the CRISPR interference-based protocol. Results using qRT-PCR indicate that co-transfection with the WT U1 snRNA elevated the amount of the 5′SS of intron 2 attached to pol II located in mid-intron 2. However, co-transfection with MUT U1 snRNA substantially increased 5′SS of intron 2 attached to pol II (Fig. 3c). In addition to the mutated 5′SS association to pol II, we examined the association of the mutated U1 snRNA to pol II located at the middle of intron 2, by RNA sequencing. In the input sample, the ratio between the exogenous MUT and the endogenous WT U1 snRNA was 1.2, reflecting overexpression efficiency. However, in the IP sample, we detected over 2-fold higher MUT U1 than WT U1 (Fig. 3d). These results indicate that the U1 snRNA-5′SS base pairing is both important for the selection of the 5′SS as well as for the tethering of the upstream 5′SS with pol II located in the downstream intron, to enable functional splicing of that intron.

### U1 snRNP and pol II are tethered to 5′SS regions of unspliced transcripts in a transcriptome-wide manner

**Fig. 4:**
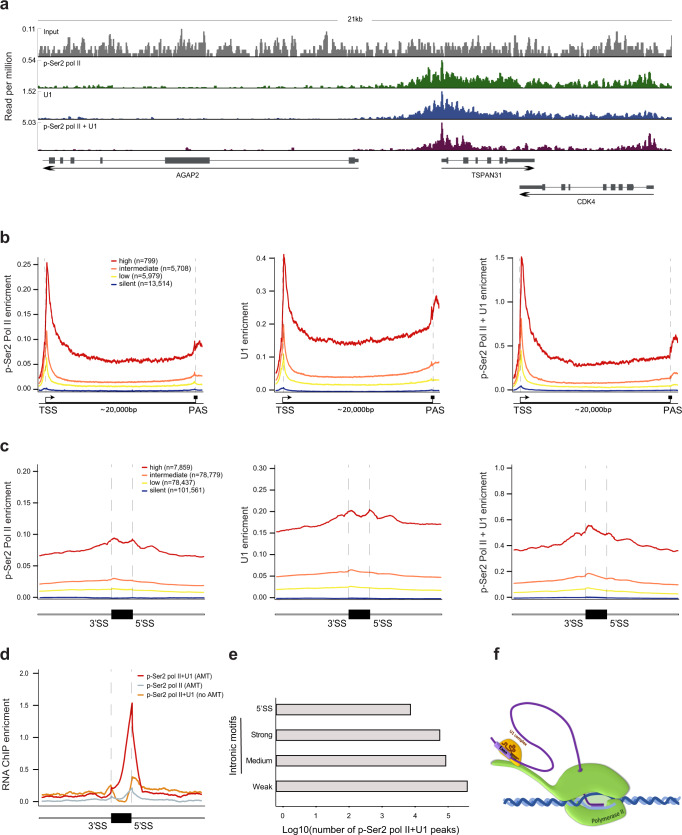
p-Ser2 pol II and U1 snRNP travel together during transcription and are linked to 5′SS regions. **a** Signals from ChIP-seq using p-Ser2 pol II antibody alone (green), U1C antibody alone (blue), or p-Ser2 pol II antibody followed by U1C antibody (purple) over a representative region of the HEK293 genome containing transcribed and untranscribed genes compared to input (gray). **b** p-Ser2 pol II, U1C, and p-Ser2 pol II-U1C occupancy over genes in HEK293 cells. Genes were divided based on expression (fpkm) into high (red), intermediate (orange), low (yellow), and silent (green). The signal is shown across 22,000 bp. Genes were scaled to 20,000 bp; 1000 bp upstream of the transcription start site and 1000 bp downstream of the polyadenylation site are shown. **c** p-Ser2 pol II, U1C, and p-Ser2 pol II-U1C occupancy over exons and 500 bp of the flanking intron sequences in HEK293 cells with genes grouped by expression level. Exons were scaled to 150 bp. One replicate for all ChIP-seq experiments. **d** RNA-ChIP profile over internal exons with 5′SS peaks and their 500 bp flanking introns in HEK293 cells for double RNA-ChIP using p-Ser2 pol II antibody followed by U1C antibody with AMT treatment (red), for single RNA-ChIP using p-Ser2 pol II antibody with AMT treatment (gray), and for double RNA-ChIP without AMT treatment (orange). Signals are normalized to input. **e** The number of pol II-U1C RNA-ChIP peaks on canonical 5′SS and on putative intronic 5′SS sites. Putative sites are divided according to their 5′SS motif strength. RNA-ChIP-seq experiments were done in duplicate. **f** Schematic model of 5′SS association with pol II via base pairing with U1 snRNA during intron synthesis.

To assess where along the genome pol II and U1 snRNP interact in living cells, we performed ChIP-seq or double ChIP-seq analyses with p-Ser2 pol II and U1C antibodies. We crosslinked HEK293 cells with FA, extracted nuclei, and fragmented DNA with sonication and MNase. After treatment with RNase A, which we used to eliminate RNA-dependent associations, we performed IPs. This method identifies DNA regions bound to elongating pol II that interact directly and indirectly with U1 snRNP. The double ChIP-seq profiles of both U1 snRNP and p-Ser2 were similar to those of ChIP-seq analyses with p-Ser2 pol II alone or U1C alone (Fig. 4a). The correlation between p-Ser2 pol II-bound and U1C-bound regions is very high (*r* = 0.988, *p* < 10^−15^, two-tailed test of Pearson’s correlation) (Supplementary Fig. 5a), indicating that pol II and U1 progress together across most expressed genes. These results are consistent with a previous study using mass spectrometry to analyze immunoprecipitates from HeLa cells that showed 90% overlap of the U1 snRNP interactome with the pol II interactome^38^, as well as with the recently reported structure of transcribing RNA polymerase II–U1 snRNP complex^18^. Comparing the ChIP-seq data with gene expression level data, revealed that U1 snRNP is associated with elongating pol II from the transcription start sites to polyadenylation sites (Fig. 4b). This result is similar to the pattern shown in experiments that used an antisense oligonucleotide-based method for U1 snRNA purification^39^. The association of U1 snRNP and p-Ser2 pol II was also shown on intronless genes^40^ (Supplementary Fig. 5b), suggesting that the interaction between U1 snRNP and p-Ser2 pol II forms independently of the splicing reaction.

Examination of all internal exons and their surrounding intronic regions revealed that U1 is associated with pol II across 5′SS regions and 3′SS regions and that levels of U1 snRNP:p-Ser2 pol II interactions were similar in exons and adjacent introns (Fig. 4c). This implies that either U1 snRNP remains associated with pol II, or that an additional U1 snRNP attaches to pol II as soon as U1 snRNP is dissociated from pol II.

To identify the 5′SS regions that are associated with the U1 snRNP and elongating pol II across the entire transcriptome, we performed RNA ChIP-seq or double RNA ChIP-seq analyses with p-Ser2 pol II and U1C antibodies. We crosslinked HEK293 cells with FA and AMT. Cells treated as above but without AMT were used as a control. After MNase digestion and sonication, we performed immunoprecipitation with an anti-p-Ser2 pol II antibody or double immunoprecipitation with the anti-p-Ser2 pol II antibody followed by an anti-U1C antibody. Next, we reversed the FA and AMT crosslinking, and the RNA was subjected to RNA-ChIP-seq. This strategy can capture the 5′SS that base paired with U1 snRNA located on the CTD of pol II or with U1 snRNA that is associated with pol II through RNA synthesized by pol II.

We observed more than 3-fold enrichment of 5′SS junctions for U1 snRNP:p-Ser2 pol II double RNA ChIP-seq with AMT treatment than for U1 snRNP:p-Ser2 pol II double RNA ChIP-seq with no AMT and for p-Ser2 pol II alone (Fig. 4d). This indicates that the AMT crosslinking, and therefore base pairing interaction of 5′SSs with U1 snRNA is required for immunoprecipitation of 5′SS with the U1 snRNP and p-Ser2 pol II. 6439 5′SS junctions were enriched for U1 snRNP:p-Ser2 pol II double RNA ChIP-seq. These 5′SS junctions were expressed at higher levels compared with the average expression level of 5′SS junctions in transcribed genes (Supplementary Fig. 5c). The 6439 5′SSs constitute 33% of all 5′SSs of expressed genes in these cells, and 60% of the 5′SSs of the highly expressed genes (Supplementary Fig. 5d). Genes that are highly transcribed have a higher probability of capture by immunoprecipitation^41^, and also to be captured by AMT crosslinking. It should be noted that double immunoprecipitation results in very low yield^42^, and that AMT crosslinking is an inefficient reaction^43^. Also, primer extension is disrupted by the inefficiency of the AMT reverse-crosslinking reaction, and the presence of psoralen monoadduct after crosslink reversal^44^. Given these technical difficulties which result in considerable down-sampling, our results strongly suggest transcriptome-wide phenomena. Among expressed genes, we identify on average 58 reads overlapping 5′SSs of the AMT crosslinked 5′SSs, compared to only 0.6 reads overlapping 5′SS of non-crosslinked 5′SS. This high coverage, which is also much larger than the intron coverage, suggests that the 58 5′SSs reads attached to U1 snRNP: elongated pol II together originated from multiple locations downstream from the 5′SSs. About ~18% of the 5′SS regions associated with U1 snRNP:p-Ser2 pol II together originated from the first intron in the gene, compared to ~15% of 5′SS in all genes (*P* < 10^−5^, one-tailed population proportion test) (Supplementary Fig. 5e, f). Enrichment of U1 sites within the first 500 nucleotides downstream of promoters was previously shown^45^. In addition, the first and the last introns are likely to be spliced out after internal introns are removed^46^. Also, 5′SSs belonging to short and long introns were associated with U1 snRNP:p-Ser2 pol II together (Supplementary Fig. 5g), and the splice-site scores of the 5′SSs associated with U1 snRNP and p-Ser2 pol II were similar to the average splice-site score in the human genome (Supplementary Fig. 5h). This analysis implies that the 5′SS attached to pol II during transcription of the downstream sequences occurs in a transcriptome-wide manner.

Apart from the bonafide 5′SS enrichment, U1 snRNP:p-Ser2 pol II together also interact with putative intronic 5′SS within expressed genes (Fig. 4e). The enrichment of U1 snRNA binding to cryptic intronic 5′SS was previously demonstrated^39^. Most of these putative intronic 5′SS are located up to 1000 bp upstream from cryptic intronic polyadenylation signals (PASs) that can activate premature polyadenylation. These results support the telescripting model^47^. Furthermore, about 19,450 exon–exon junctions in expressed genes were enriched for U1 snRNP and p-Ser2 pol II together (compared to 6439 5′SS junctions). These results show that the U1 snRNP and p-Ser2 pol II are tethered together to both spliced and unspliced junctions, and indicate that the majority of the time a transcript is attached to elongating pol II is in its spliced form.

We next measured the levels of all spliceosomal snRNAs associated with the U1 snRNP and p-Ser2 pol II. Only U1 snRNA was significantly enriched (Supplementary Fig. 5i). This finding suggests that precipitation of the U1 snRNP:p-Ser2 pol II together captures only the early stages of spliceosome assembly. Taken together, these findings support our model that intron looping that results from interactions between the 5′SS and U1 snRNA and U1 snRNA and pol II facilitate rapid co-transcriptional splicing.

## Discussion

Here, using a CRISPR interference-based approach to pause pol II transcription in the middle of introns, we discovered that the nascent 5′SS base pairs with a U1 snRNA tethered to pol II during intron synthesis. These associations are functional and occur across the transcriptome. Since the upstream 5′SS remains attached to the transcriptional machinery an intron loop can form during transcription, bringing the 5′SS into proximity with the 3′SS. We speculate that this spatial proximity facilitates the rapid splicing observed in mammalian cells in vivo even for genes with very long introns.

The association of U1 snRNP with the transcription machinery may be related to additional functions such as telescripting (in which U1 snRNP binds to putative intronic 5′SSs and prevents activation of premature polyadenylation sites), transcription initiation, capping, polyadenylation^48^, and to the mobilization of lncRNAs to nearby genes or regulatory sites within their proximal or distal chromatin regions^49^.

Our results indicate that exon 2 of *FRG1* is selected via exon definition. The 5′SS of that exon is important for U2 snRNP binding to the branch site of intron 1, implying that a cross-exon complex is formed. However, the same 5′SS is associated with pol II during the downstream intron synthesis in a fashion that resembles intron definition^3^. The explanation for this discrepancy is that cross-exon interactions during the exon-definition mode are later converted to a cross-intron complex, i.e., the commitment complex^6,50^. Thus, U1 snRNP that binds to the 5′SS can have a dual role, first in cross-exon definition and then, as pol II transcribes the downstream intron, to facilitate the formation of a cross-intron complex.

Our results imply that during exon definition, the advancement from the commitment complex to complex A, in which U2 snRNP binds the branch site, requires that U1 snRNP binds to the downstream 5′SS. In contrast, Nojima et al.^10^ showed using mNET-seq analysis, high intermediate signal when p-ser5 pol II was accumulated over downstream exon. In addition, Reimer et al^20^ pointed out that for 75% of mammalian introns splicing is achieved when pol II is located within ~300 nucleotides downstream from the 3′SS. As exons are about 150 nucleotides long^51^, this implies that for most of the introns splicing is carried out during exon synthesis, and presumably independently of the synthesis of the downstream 5′SS. This suggests that those introns are spliced via intron definition, while for other introns the synthesis of the downstream 5′SS is required for splicing via exon definition.

There is evidence suggesting that only one U1 snRNP is associated with pol II. In a cryo-EM structure of a complex between purified pol II and purified U1 snRNP assembled in vitro, the ratio is 1:1^18^. This implies that only one 5′SS can be handled each time. In this situation, when only one U1 snRNP handles splicing of all introns in a multi-intron gene, the 5′SS must be handed over from U1 to U5 and U6 snRNPs during exon synthesis, in order to advance spliceosome assembly from the commitment complex to complex B^6,17^. Under these conditions, the same U1 snRNP is free to bind to the downstream 5′SS. However, our data suggest that two or more unspliced 5′SSs can be attached to pol II. Introns are not removed in a “first come first serve” manner, meaning that some 5′SSs are spliced only after the synthesis of several additional 5′SSs located downstream along the gene^46,52–54^. In addition, intron splicing order has been shown to influence alternative splicing in *COL5A1*^55^, and intron splicing order can affect splicing fidelity^56,57^. This implies that for certain genes, several 5′SSs and apparently several U1 snRNP, are associated with pol II, and following the formation of the commitment complex, the advance to a higher order of spliceosome assembly can be delayed until a downstream signal is obtained. The association of several unspliced 5′SSs and 3′SSs of different introns of the same gene along the CTD of pol II may also explain how the back-splicing reaction can occur^7^.

The CTD of pol II and splicing factors are incorporated into phase-separated condensates in a process mediated by CTD phosphorylation^15^. The confinement of the growing intron, regardless of its length, within a transcription droplet might reduce the physical forces imposed on the base pairing between U1 snRNA and the 5′SS during intron synthesis. This might explain why during human evolution introns lengthened without compromising the splicing fidelity^23^.

Our results support a model that explains how exon definition occurs co-transcriptionally, rapidly, and independently of intron length in vivo (Fig. 4f). During transcription, U1 snRNP and U2AF2 bind the pol II CTD, and the elongating pol II remains associated with the 5′SS throughout synthesis of an intron via base-pairing interaction with the U1 snRNA. Transcription elongation is slowed at the 3′SS and the pol II CTD-bound U2AF2 binds the PPT, bringing the two ends of the intron together to form the commitment complex. Upon synthesis of the downstream 5′SS the same or another U1 snRNP located on the CTD of pol II binds the downstream 5′SS, and together with U2AFs bound to the upstream PPT-3′SS region, facilitates binding of U2 snRNP to the upstream branch site. Thus, pol II plausibly assists in the formation of the commitment complex and in the selection of exons. In summary, our data imply that the initial step of spliceosome assembly occurs rapidly because the transcription machinery brings the upstream 5′SS into close spatial proximity of the 3′SS.

## Methods

### Cell maintenance and minigene construction

Flp-In-HEK293 (Invitrogen), HEK293 (ATCC), and HeLa (ATCC) cells were cultured in complete DMEM medium (Biological Industries Israel), 10% fetal bovine serum (Biological Industries Israel), 2 mg/ml l-alanyl-l-glutamine (Biological Industries Israel), 100 U/ml penicillin and 0.1 mg/ml streptomycin (Biological Industries Israel) at 37 °C in a humidified atmosphere with 5% CO_2_.

### Cloning

The *FRG1* minigene (exons 4–6 genomic region of the endogenous gene) was amplified from human genomic using primers containing restriction enzymes *Kpn*I and *Bam*HI (Supplementary Table 2). The PCR product and the vector were digested and ligated into the vector pcDNA™5/FRT/TO (Invitrogen). The clone was verified by sequencing. For stable cell lines the plasmids were integrated into Flp-In-HEK293 cells using the Flp-In system according to the manufacturer’s instructions. U1 WT plasmid (a gift from Prof. Alan M. Weiner) was re-cloned into pcDNA3.1 plasmid using BamHI.

### sgRNA design and cloning

sgRNA expression plasmid pX552 was cut with SapI (NEB). sgRNA sequences were designed using the CRISPR Design Tool (http://crispr.mit.edu/). Each sgRNA was designed together with its complement sequence, and 3 nt, ACC or CAA, were added to the 5′ end of each, respectively, to complement the *Sap*I restriction site. sgRNA and sgRNA_complement oligonucleotides were annealed followed by phosphorylation at the 5′ end using T4 Polynucleotide Kinase (NEB) (thermo-cycling program: 30 min at 37 °C PNK reaction, 20 min at 65 °C heat inactivation, 5 min at 95 °C denaturation and ramp to 4 °C at 0.5 °C per sec to anneal). For each ligation reaction, sgRNAs were diluted 1:25. Ligations of sgRNA inserts with linearized pX552 were done using T4 ligase (NEB) according to the manufacturer’s instructions. A 2-µl aliquot of the ligation reaction was used to transformation into *Escherichia coli* XL-10 Gold strain after heat shock. Positive colonies were confirmed by Sanger sequencing. sgRNAs sequences are listed in Supplementary Table 1.

### Site-directed mutagenesis

Overlapping oligonucleotide primers containing the desired mutations were used to amplify the *FRG1* MUT minigene plasmid (position + 1G to A), the *FRG1* MUTx2 minigene plasmid (positions + 3A to G and +6T to A), and the U1 MUT gene (with compensatory mutations to *FRG1* MUTx2) using KAPA HiFi HotStart (KAPA Biosystems) according to manufacturer’s instructions. After PCR amplification, *Dpn*I (New England Biolabs) digestion was done for 1 h at 37 °C. The *Escherichia coli* XL-10 Gold strain was transformed with 1–3 μl of the reaction, and colonies were picked for Mini-prep extraction (Geneaid). Sequencing confirmed that the plasmids carried the desired mutations.

### Transfection

HEK293 cells and cells that stably express either WT or MUT *FRG1* minigenes were transfected with 3 µg of 2 sgRNAs expressions plasmid pX552 located in close proximity for each intron together with 3 µg of HA–dCas9 expression plasmid (a gift from Dr. Mazhar Adli^58^). sgRNAs sequences are listed in Supplementary Table 1.

HEK293 cell lines were transfected with 3 µg of a plasmid for expression of U1 WT or U1 MUT together with 3 µg of an expression plasmid of the *FRG1* MUTx2. All transfections were done using TransIT®-LT1 transfection reagent (Mirus) according to manufacturer instructions. Experiments were performed 48 h after transfection.

### Antisense oligonucleotide treatment

The cell line that stably expresses WT *FRG1* was treated with 750 nM of a 2′ *O*-methyl-RNA oligonucleotide (IDT) using Lipofectamine 2000 (Invitrogen) according to manufacturer’s instructions. The oligonucleotide sequence is 5′-CAGCACUUACAUUUUGAAAG-3′. Experiments were performed 48 h after transfection.

### Small interfering RNA (siRNA) treatment

The cell line that stably expresses WT *FRG1* was treated with 200 nM non-targeting pool siRNA (Dharmacon; D-001810-10-05) and 150 or 200 nM U2AF65 siRNA (Dharmacon)^59^ using RNAiMAX (Invitrogen) according to manufacturer’s instructions. The siRNA sequence is 5′-GCACGGUGGACUGAUUCGUdTdT-3′. Experiments were performed 48 h after transfection.

### Co-transcriptional splicing

HEK293 cells were fractionated according to Amy Pandya-Jones protocol^29^. The fractionation was assessed by western blot using antibodies to α-tubulin (ab18251) (1:40,000), U1C (ab157116) (1:200), and histone H4 (Millipore; 05-858) (1:30,000) proteins. Chromatin-associated RNA was extracted using Trizol reagent (Sigma) and cDNA synthesis was performed with random primers according to RT-FLEX (Quanta) manufacturer’s instructions. qPCR was performed with primers located on exon–exon junction and exon–intron junction (Supplementary Table 2).

### Co-immunoprecipitation

Approximately 10 × 10^6^ cells per sample were trypsinized, washed with PBS, and crosslinked with 1% FA at 37 °C for 10 min. The reaction was quenched by addition of glycine (0.125 M) and incubated at 37 °C for 5 min. Samples were centrifuged at 2300 × *g*, washed with cold PBS, and centrifuged again. Nuclei isolated as described in ref. ^60^. Cells’ pellet was suspended in buffer 1 (60 mM KCl, 15 mM NaCl, 5 mM MgCl_2_, 0.1 mM EGTA, 15 mM Tris-HCl [pH 7.5]) supplemented with 0.5 mM DTT, 0.1 mM PMSF, 1× complete protease inhibitor (CPI), and incubated in 0.2% IGEPAL CA-630 (NP-40). After incubation for 10 min, residual NP-40 was cleared by centrifugation on a 1.2 M sucrose cushion. Nuclei were suspended in MNase digestion buffer (0.32 M sucrose, 50 mM Tris-HCl [pH 7.5], 4 mM MgCl_2_, 1 mM CaCl_2_) supplemented with 0.1 mM PMSF. MNase (10 U/10^6^ nuclei, Worthington) was added, and samples were incubated at 37 °C for 10 min. The reaction was stopped by the addition of 1 mM EDTA. Nuclei were then sedimented by centrifugation, and nuclei were used for experiments. MNase-digested nuclei were suspended in immunoprecipitation (IP) buffer (50 mM HEPES [pH 7.6], 500 mM LiCl, 1 mM EDTA, 0.7% DOC, 1% NP-40, 0.1% SDS, 1× CPI) and rotated for 1 hr at 4 °C. The samples were sonicated using a Bioruptor (Diagenode) at 40% amplitude in intervals of 2.2 s pulses with 9.9 s pauses for 10 min, followed by centrifugation 10,000 × *g* for 10 min. This supernatant is denoted as “input”. Antibodies (6 μg) used for immunoprecipitation were anti-pol II p-Ser2 (Abcam; ab5095), anti-pol II p-Ser5 (Abcam; ab5408), and anti-IgG (Santa Cruz Biotechnology; sc2027). Antibodies were added to each input sample and rotated overnight at 4 °C. Mix of protein A and G beads (50 μl, Dynabeads Invitrogen) were washed and added to each sample and rotated for 4 h at 4 °C. Beads were washed four times with IP buffer and once with 0.5 ml RNase A buffer (PBS, 0.02% Tween 20, CPI, 0.1 mM PMSF). Samples were re-suspended in 450 μl RNase A buffer and 1 μl of 10 mg/ml RNase A (Sigma-Aldrich) and incubated for 30 min at 37 °C. All samples were washed another three times with 0.5 ml IP buffer. Protein was eluted from the beads by adding 100 μl PBS and 20 μl 6× SDS sample buffer (272 mM Tris-HCl [pH 6.8], 30% glycerol, 12% SDS, 20% β-mercaptoethanol, 0.01% bromophenol blue) and incubating in a thermo-shaker for 15 min at with vigorous shaking. The supernatant was moved to a new tube, reversed crosslink at 75 °C for 1 h, and boiled for 5 min at 100 °C.

### Western blots and antibodies

Proteins were separated by SDS-PAGE on 8% or 10% polyacrylamide gels and transferred to 0.45-μm nitrocellulose membranes (Whatman Protran). The membranes were incubated with the appropriate primary and secondary antibodies and washed with TBS-Tween 20. Horseradish-peroxidase-conjugated secondary antibodies were detected by SuperSignal West Pico Chemiluminescent Substrate (Thermo Scientific; PI-34080). Antibodies used were anti-pol II p-Ser2 (Abcam; ab5095) (1:500), anti-pol II p-Ser5 (Abcam; ab5408) (1:1000), anti SNRPC (U1C) (Abcam; ab157116) (1:200), anti-U2AF2 (a gift of Prof. Juan Valcarcel, Centre for Genomic Regulation, Barcelona, Spain) (1:500), anti-U2AF35 (Abcam; ab172614) (1:250), anti-FUS (Abcam; ab23439) (1:400), anti-SAP155/SF3B1 (MBL; D221-3) (1:1000), anti-NXF1/TAP (Santa Cruz Biotechnology; sc- 32319) (1:500), anti-GAPDH (GenScript; A00191-40) (1:1000), anti-PTBP1 (Abcam; ab133734) (1:5000), anti a-tubulin (abcam; ab18251) (1:40000), anti-histone 4 (Millipore;05-858) (1:30000), donkey anti-rabbit IgG (Abcam; ab97064), and goat anti-mouse IgG (Abcam; ab7068).

### RNA-ChIP

RNA-ChIP was performed using the RNA ChIP-IT kit (Active Motif) as detailed in the manufacturer’s instructions with the following minor modification: cells from three 10-cm plates were harvested, placed on ice, and crosslinked with 1% FA (Sigma) followed by crosslinking with 0.2 mg/ml AMT (Sigma; A4330) by irradiation with 350 nm UV light (Vilber Lourmat ECX.F20.L; 7 mW/cm^2^) for 45 min. After isolation of nuclei (as describe in co-immunoprecipitation), 500 µl of MNase buffer (0.32 M sucrose, 50 mM Tris-Cl, pH 7.5, 4 mM MgCl_2_, 1 mM CaCl_2_, and 0.1 mM PMSF) was added. Chromatin and RNA were digested using 150 U of MNase (Worthington) for 15 min at 37 °C with shaking at 400 RPM on a thermomixer. The enzyme was inactivated by adding 0.8 mM EDTA. The samples were centrifuged at 12,000 × *g* for 5 min, and the nuclei were sonicated using a Bioruptor at 40% amplitude with intervals of 2.2 s pulses with 9.9 s pauses for 10 min. This yielded DNA and RNA fragments of 100–500 bp in size. Antibodies used for immunoprecipitation were anti-pol II p-Ser2 (Abcam; ab5095), anti-pol II p-Ser5 (Abcam; ab5408), anti-U2 snRNP A (B-3) X (Santa Cruz; sc-393804X), anti-U2AF2 (a gift of Prof. Juan Valcarcel, Centre for Genomic Regulation, Barcelona, Spain), and anti-HA (Abcam; ab9110). Following elution and proteinase K digestion the crosslinks were FA reversed by 65 °C for 1.5 h and AMT reversed by irradiation with 254 nm UV light for 10 min with samples on ice. RNA was extracted using Trizol-LS reagent (Invitrogen). For DNA extraction, samples were eluted with elution buffer (0.5% SDS, 300 mM NaCl, 5 mM EDTA, 10 mM Tris-HCl, pH 8.1), digested with RNase cocktail (Invitrogen) and RNase H (NEB) for 30 min at 37 °C following proteinase K and reverse crosslinking at 65 °C. Extraction was done using phenol:chloroform:isoamyl alcohol (Sigma).

### cDNA synthesis and qPCR

cDNA synthesis was performed with RT-FLEX (Quanta) according to the manufacturer’s instructions. qPCR was performed using KAPA SYBR FAST Universal qPCR kit (KAPA Biosystems) according to the manufacturer’s instructions. Primers are listed in Supplementary Table 2.

### RNA-seq

RNA was extracted from HEK293 cells using Trizol reagent (Sigma). Deep sequencing libraries were prepared using TruSeq Stranded mRNA library preparation kits as per the manufacturer’s instructions. Sequencing of 125-bp paired-end reads was performed using an Illumina HiSeq 2000.

### Double ChIP-seq

ChIP was performed as described previously^61^ with the following modifications: Approximately 7 × 10^6^ HEK293 cells were used per sample. After nuclei purification and MNase digestion as described in co-immunoprecipitation, samples were sonicated using a Bioruptor at 40% amplitude with intervals of 2.2 s pulses with 9.9 s pauses for 12 min. For anti-pol II p-Ser2 immunoprecipitation, 80 µl of protein A and G Dynabeads (Invitrogen) mixture was combined used with 18 µg anti-pol II p-Ser2 antibody (Abcam; ab5095) and added to the sample. After immunoprecipitation, 1 µl of 10 mg/ml RNase A (Sigma) was added, and samples were incubated for 30 min at 37 °C. Following washes, the samples were eluted with 50 μl fresh 0.1 M DTT and incubated at room temperature for 5 min. As previously described^44^, 50 µl of freshly prepared 2X Chromatin Release Buffer (500 mM NaCl, 2% deoxycholate, 2% SDS, 2 mM EDTA) with fresh EDTA­free protease inhibitor cocktail and PMSF (from the RNA ChIP-IT kit) were added, and samples were incubated at 37 °C for 55 min. The elution step was repeated, and samples were incubated for 30 min at 37 °C. This step releases bound chromatin and inactivates the antibodies used in the first ChIP. One-half of the eluted samples were treated with 1.5 µl Proteinase K (NEB), and incubated for 16 h at 65 °C. DNA was purified using phenol:chloroform:isoamyl alcohol (Sigma) extraction. The other half of eluted samples were subjected for the second ChIP, 50 µl of protein A and G Dynabeads (Invitrogen) was combined with 10 µg anti-U1C antibody (Abcam; ab157116) and added to the samples. After incubation of 16 h at 4 °C, the samples were washed and eluted as described previously^61^. Deep sequencing libraries were prepared using Illumina TruSeq library preparation kits as per the manufacturer’s instructions. Sequencing of 50-bp single-end reads was performed using an Illumina HiSeq 2000.

### Double RNA-ChIP-seq

Double RNA-ChIP was performed using the RNA ChIP-IT kit (Active Motif) as detailed in the manufacturer’s instruction with the following modifications: First, 9 × 10^7^ cells were used. For the first RNA-ChIP, 700 µl of protein A and G Dynabeads mixture (Invitrogen) was combined with 90 µg anti-pol II p-Ser2 antibody (Abcam; ab5095). After washes, the samples were eluted with 150 μl fresh 0.1 M DTT and incubated at room temperature for 5 min. Next, as described previously^44^, 150 µl of freshly prepared 2× Chromatin Release Buffer with protease inhibitor cocktail, PMSF, and RNase inhibitor (from the RNA ChIP-IT kit) were added and mixed well. The samples were incubated at 37 °C for 55 min. The elution step was repeated, and samples were incubated for 30 min at 37 °C. The eluted samples were divided into two aliquots. One aliquot of 300 µl was kept at −80 °C until the second ChIP step. To the other 300 µl aliquot, 2 µl 5 M NaCl and 2 µl proteinase K (from the RNA ChIP-IT kit) were added, and the samples were incubated at 42 °C for 1 h to digest the proteins and then at 65 °C for 1.5 h to reverse FA crosslinking. The AMT crosslinks were reversed by irradiation with 254 nm UV light for 10 min with samples kept on ice. The RNA was extracted using Trizol reagent (Sigma). For the second RNA-ChIP, 150 µl protein A and G Dynabeads (Invitrogen) mixture was combined with 40 µg anti-U1C antibody (Abcam; ab157116) and added to the samples. Samples were incubated for 16 h at 4 °C. Washes and the elution were done as described in the instructions for the RNA ChIP-IT kit. A no-AMT experiment was used for control. Deep sequencing libraries were prepared using v2-pico (Takara Bio) library preparation kits. Sequencing of 75 bp paired-end reads was performed using an Illumina HiSeq 2000.

### RNA-seq, ChIP-seq, and RNA ChIP-seq alignment

To improve read quality for RNA-seq, paired reads were trimmed to keep only the first 100 bases prior to alignments. For RNA ChIP-seq, duplicate experiments were combined. The last 3 bps of each single-end read were trimmed and TruSeq barcodes were removed using trimmomatic software version 0.39^62^ prior to alignment. Sequenced reads were aligned to the human genome (Assembly hg38, GRCh38 Genome Reference Consortium Human Reference 38) using Bowtie2 v2.1.0^63^ for reads derived from DNA fragments and using STAR aligner v2.7.1a^64^.

### ChIP-seq and RNA ChIP-seq occupancy

The sequencing coverage and depth were represented using the UCSC bigWig format. Sequencing depth files at single-base resolution were created using bam2wig.pl tool (http://search.cpan.org/∼tjparnell/Bio-ToolBox-1.44/) and for each base, a normalized reads-per-million value was calculated considering all the reads that span that base. The normalized input coverage was subtracted from the normalized ChIP-seq and RNA-seq coverage to represent the difference between the two samples and the final occupancy value for each base. Data stored in bigWig format was extracted using bwtool^65^ fed with relevant BED files. The bwtool was also used to align features to start or end coordinates and to calculate the mean single-base values for multiple features in a given BED file. Exon–intron junctions that were enriched in AMT pol II-U1 conditions were identified using an in-house Perl script. A 5′SS was defined as a peak if there was at least one read overlapping the exon–intron junction and in addition, there was at least a 1.5-fold enrichment in the normalized (RPM) reads compared to RNA-ChIP input. Putative intronic 5′SS peaks were called based on the 1.5-fold-change difference in the region 200 bp upstream to the site relative to input. For intronic peaks, every intron was split to 200 bp regions and each region was tested for a 1.5-fold difference from input, and subsequently, adjacent peak regions were merged.

### Gene expression and isoform abundance calculation

RNA quantification was performed using Cufflinks v2.2.1^66^ fed with RefSeq genes table and with default parameters. For DNA-ChIP analysis gene expression was defined as high, intermediate, low, or silent based on whether fragments per kilobase per million (fpkm) values were greater than 100, between 10 and 100, between 1 and 10, or below 1, respectively. For RNA-ChIP analyses, genes were defined as expressed if they had an fpkm value greater than 1.

### Splice-site strength scores

We used MaxEntScan: score 5′SS^67^ using the maximum entropy model to calculate the strength of the 5′SS for each exon in our RefSeq exons table. For analysis of cryptic sites, all 9mers around intronic GT sites were defined as strong, medium, and weak if their score were above 8.77, 7.39, and 4, respectively, and the rest of GT sites were discarded following Almada et al^45^.

### U1 mutant and wild-type read counts

To identify reads originated from the WT versus MUT U1, sequenced reads were aligned to a customized reference genome comprised of U1 WT and MUT sequences using Bowtie2 v 2.4.2^68^ with default parameters as well as: --fr --no-discordant --no-unal -X 164 --no-mixed. Reads were then counted within the mutated region (see Site-directed mutagenesis) using the bam signals R package (https://bioconductor.org/packages/release/bioc/vignettes/bamsignals/inst/doc/bamsignals.html).

### Reporting summary

Further information on research design is available in the Nature Research Reporting Summary linked to this article.

## Supplementary information

Supplementary Information

Reporting Summary

## Data Availability

The data that support this study are available from the corresponding author upon reasonable request. All ChIP-seq, RNA ChIP-seq, and RNA-seq raw and processed data generated in this study have been deposited in and are publically available in the GEO database under accession code GSE145092. Source data are provided with this paper.

## Source data

Source Data
