# Supplementary material for: The upstream 5′ splice site remains associated to the transcription machinery during intron synthesis
Source: Nat Commun. 2021 Jul 27;12:4545. doi: 10.1038/s41467-021-24774-6 (PMC8316553; doi:10.1038/s41467-021-24774-6)
Supplement: Supplementary file 1 — Supplementary Information [file 41467_2021_24774_MOESM1_ESM.pdf]

SUPPLEMENTARY INFORMATION

The upstream 5' splice site remains associated to the transcription machinery during intron synthesis

Yodfat Leader[1*], Galit Lev Maor[1*†], Matan Sorek[2*], Ronna Shayevitch[1], Maram Hussein[1], Ofir Hameiri[1], Luna Tammer[1], Jonathan Zonszain[1], Ifat Keydar[1], Dror Hollander[1], Eran Meshorer[2], and Gil Ast[1 †]

[1]Department of Human Molecular Genetics and Biochemistry, Sackler Faculty of Medicine, Tel-Aviv University, Ramat Aviv 69978, Israel.

[2]Department of Genetics, The Institute of Life Sciences, and The Edmond and Lily Center for Brain Sciences (ELSC). The Hebrew University of Jerusalem, Edmond J. Safra Campus, Jerusalem 91904, Israel.

[*]These authors contributed equally to this work.

[†]Corresponding authors. Contact information: Gil Ast, gilast@post.tau.ac.il. Galit Lev Maor, galitlm@tauex.tau.ac.il

Supplementary Figures

Supplementary Figure 1

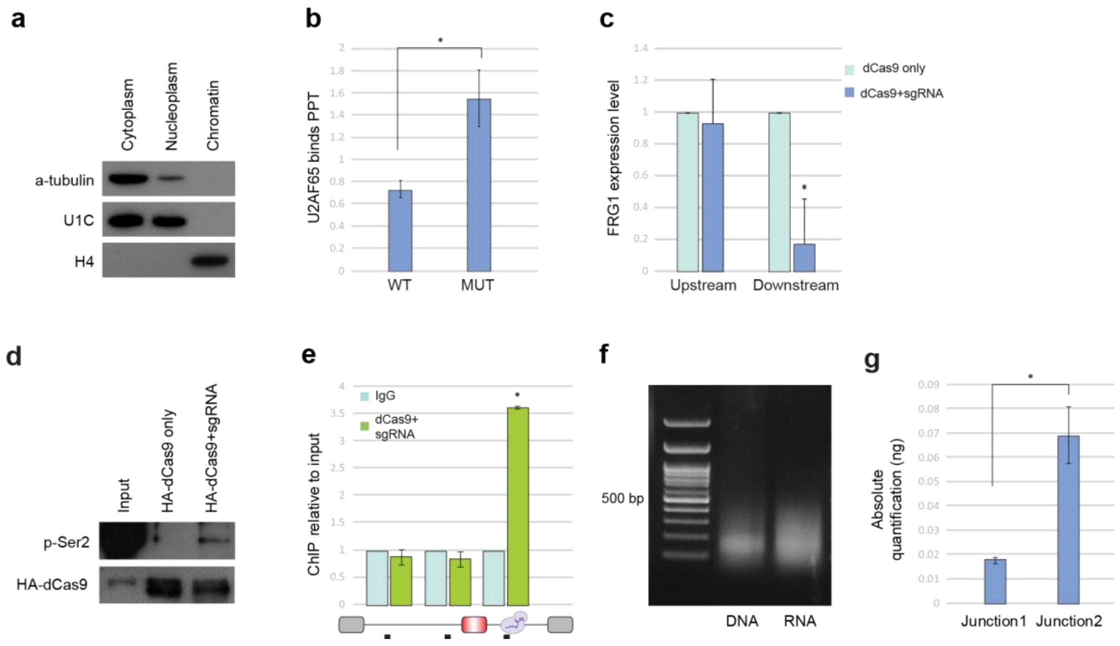

Supplementary Figure 1. CRISPR- interference analyses. a. HEK293 cells fractionation. a western blot was done with anti-α-tubulin, anti-U1C, and anti-Histone 4 antibodies detected in the cytoplasmic, nucleoplasmic, and chromatin fractions, respectively. Twenty-five micrograms of total protein from each fraction was loaded per lane. Source data are provided as a Source Data file. **b.** *FRG1* WT and MUT cells were crosslinked with FA and nuclei were isolated. RNA was fragmented with MNase and sonication. Immunoprecipitation was performed using U2AF65 antibody, the RNA was extracted, and qRT-PCR was performed to examine the enrichment of the PPT of intron 1 *FRG1* minigene. N=3 independent experiments. Error bars show mean values +/- SEM. Asterisk indicates P = 0.037 two-tailed t-test. **c.** *FRG1* WT cells were transfected with a plasmid for expression of HA-dCas9 with or without the plasmid for expression of sgRNA targeted against a location in the middle of *FRG1* intron 2. After 48 h RNA was extracted and RT was done using hexamers primer. Relative *FRG1* RNA level were measured using qRT-PCR with primers specific for nascent *FRG1* transcript. The primers were located near the start and end of the transcript. N=3 independent experiments. Error bars show mean values + SEM. Asterisk indicates P = 0.037 two-

tailed t-test. **d.** The CRISPR interference-based protocol was performed. After HA-dCas9 IP, a western blot was done with anti-p-Ser2 pol II and anti-HA antibodies. Source data are provided as a Source Data file. **e.** CRISPR interference-based protocol targeting the middle intron of the *FRG1* minigene was performed, and mean DNA levels were measured by qPCR. *N*=3 independent experiments. Error bars show mean values +/- SEM. Asterisk indicates *P* = 0.020 two-tailed t-test. Each bar corresponds to the amplified segment marked in the gene diagram below the graph. The sgRNA binding is indicated as a purple shape. **f.** Agarose gel of purified DNA and RNA after MNase and sonication treatment. **g.** RNA extraction from WT cells CRISPR interference-based protocol. RNA was quantified by absolute qRT-PCR. *N*=3 independent experiments. Error bars show mean values +/- SEM. Asterisk indicates *P* = 0.032 two-tailed t-test. Junction-1 denotes the exon 1- intron 1 junction, and Junction-2 denotes the exon 2-intron 2 junction.

Supplementary Figure 2

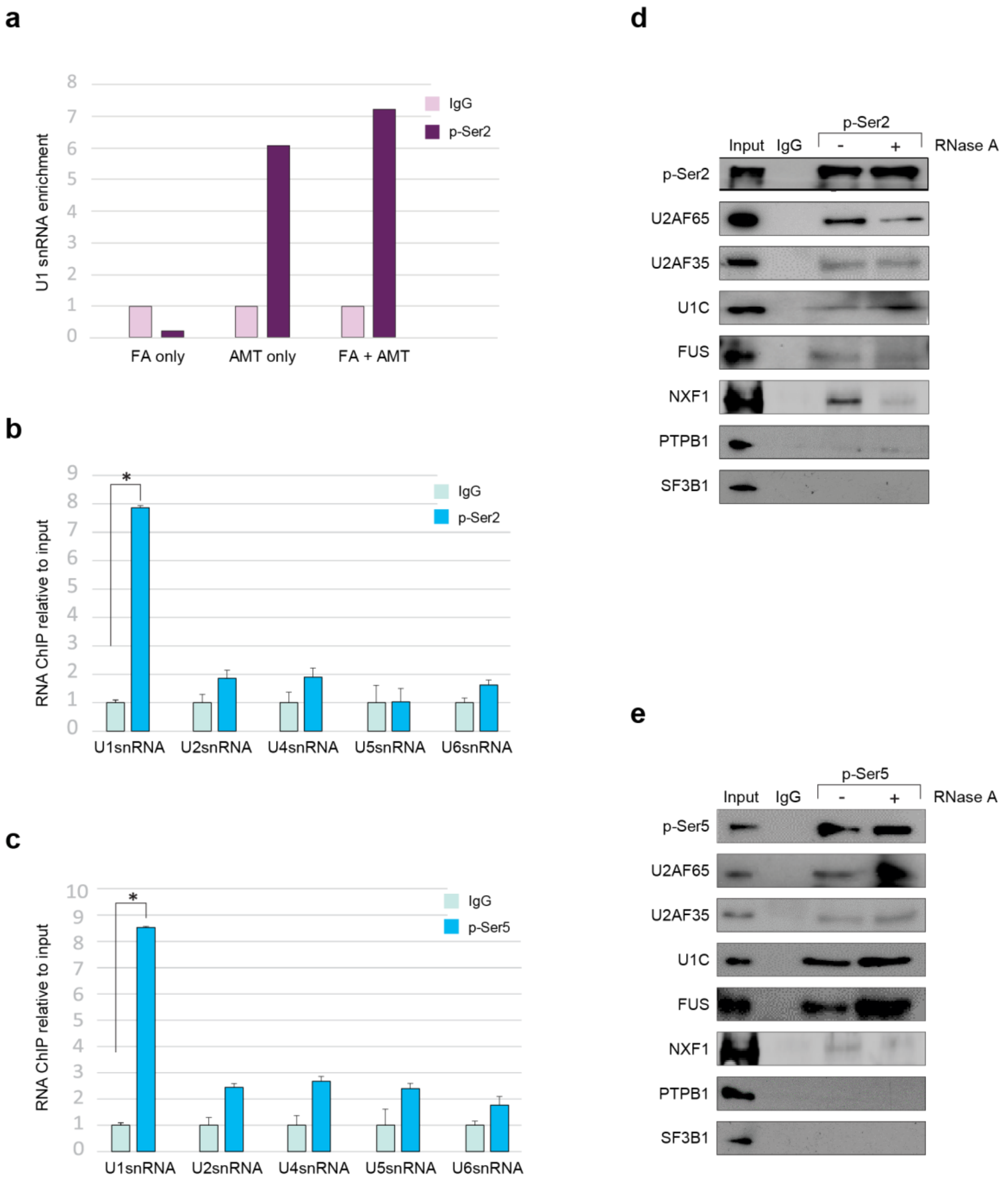

Supplementary Figure 2. U1 snRNP associates with pol II. **a.** HEK293 cells were either crosslinked or not with FA and AMT, followed by purification of nuclei, sonication, and RNA-ChIP using p-Ser2 pol II antibody or IgG antibody as a control. Total RNA was extracted and reverse transcribed using RT-FLEX with hexamer primers. U1 snRNA was quantified by qRT-PCR in one experiment. **b, c.** HEK293 cells were treated with FA and AMT, followed by UV irradiation, purification of nuclei, and

DNA and RNA fragmentation, and RNA-ChIP with antibodies against: p-Ser2 **(b)** and p-Ser5 **(c)** pol II CTD. snRNA levels were measured by qRT-PCR. N=4 for b and N=3 for c independent experiments. Error bars show mean values + SD. Asterisk indicates for panel b P = 1.23x10^{-6}, for panel c P = 4.7x10^{-}4, two-tailed t-test. **d, e.** Nuclear extracts of HEK293 cells were treated or not with RNase A and subjected to immunoprecipitation with antibodies against: p-Ser2 **(d)** and p-Ser5 **(e)** pol II CTD. Western blotting was performed with the indicated antibodies. Source data are provided as a Source Data file.

Supplementary Figure 3

a

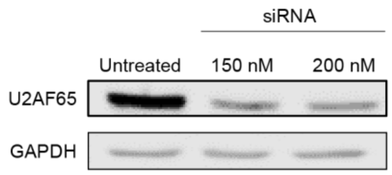

b

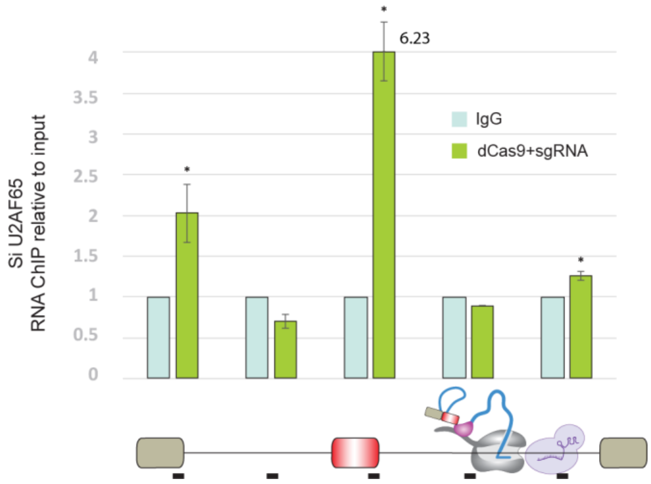

Supplementary Figure 3. U2AF2 and the PPT are not essential for 5'SS association with pol II. a. Western blot analysis of total protein extracted 48 h after U2AF2 KD using 150 nM or 200 nM of siRNA. Source data are provided as a Source Data file. **b.** CRISPR interference-based experiment was performed after U2AF65 siRNA to evaluate the association of various transcript regions with pol II located mid-intron 2 of WT *FRG1* minigene. Mean RNA levels were measured. N=3 independent experiments. Each bar corresponds to the amplified segment marked in the gene diagram below the graph. Error bars show mean values +/- SEM. From left to right asterisk indicates P = 0.05, P = 0.007, P = 0.002, two-tailed t-test.

Supplementary Figure 4

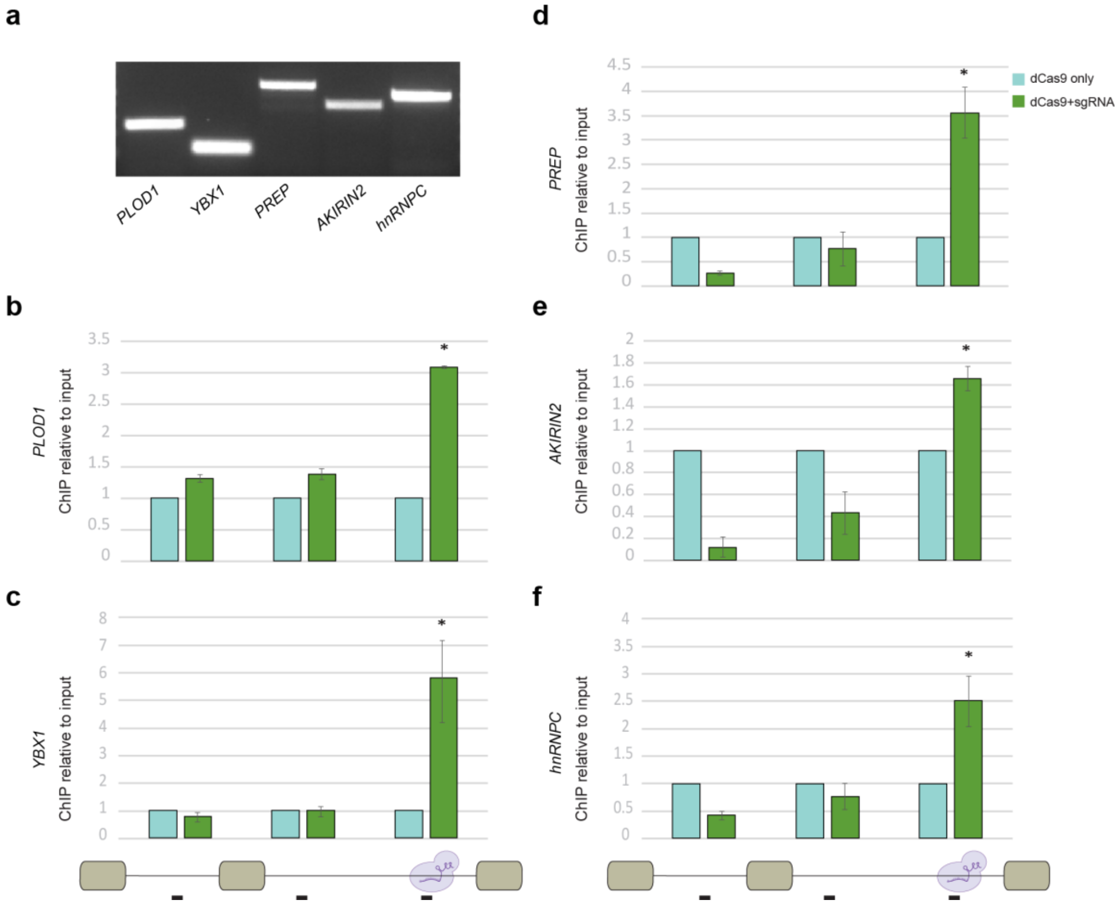

Supplementary Figure 4. Splicing pattern of the endogenous genes, and sgRNA binding efficient. a. Total RNA was extracted from HEK293 cell line. RT-PCR was performed to examine splicing pattern of the selected three exons. Source data are provided as a Source Data file. **b-f.** Binding specificity of the sgRNAs. CRISPR interference-based protocol targeting the middle intron of the indicated genes was performed, and mean DNA levels were measured by qPCR. N=3 independent experiments. Error bars show mean values +/- SEM. Asterisk indicates for panel b P = 4.4x10^{-8}, for panel c P = 0.016, for panel d P = 0.008, for panel e P = 0.004, for panel f P =0.03, two-tailed t-test. Each bar corresponds to the amplified segment marked in the gene diagram below the graph. The sgRNA binding is indicated as a purple shape.

Supplementary Figure 5

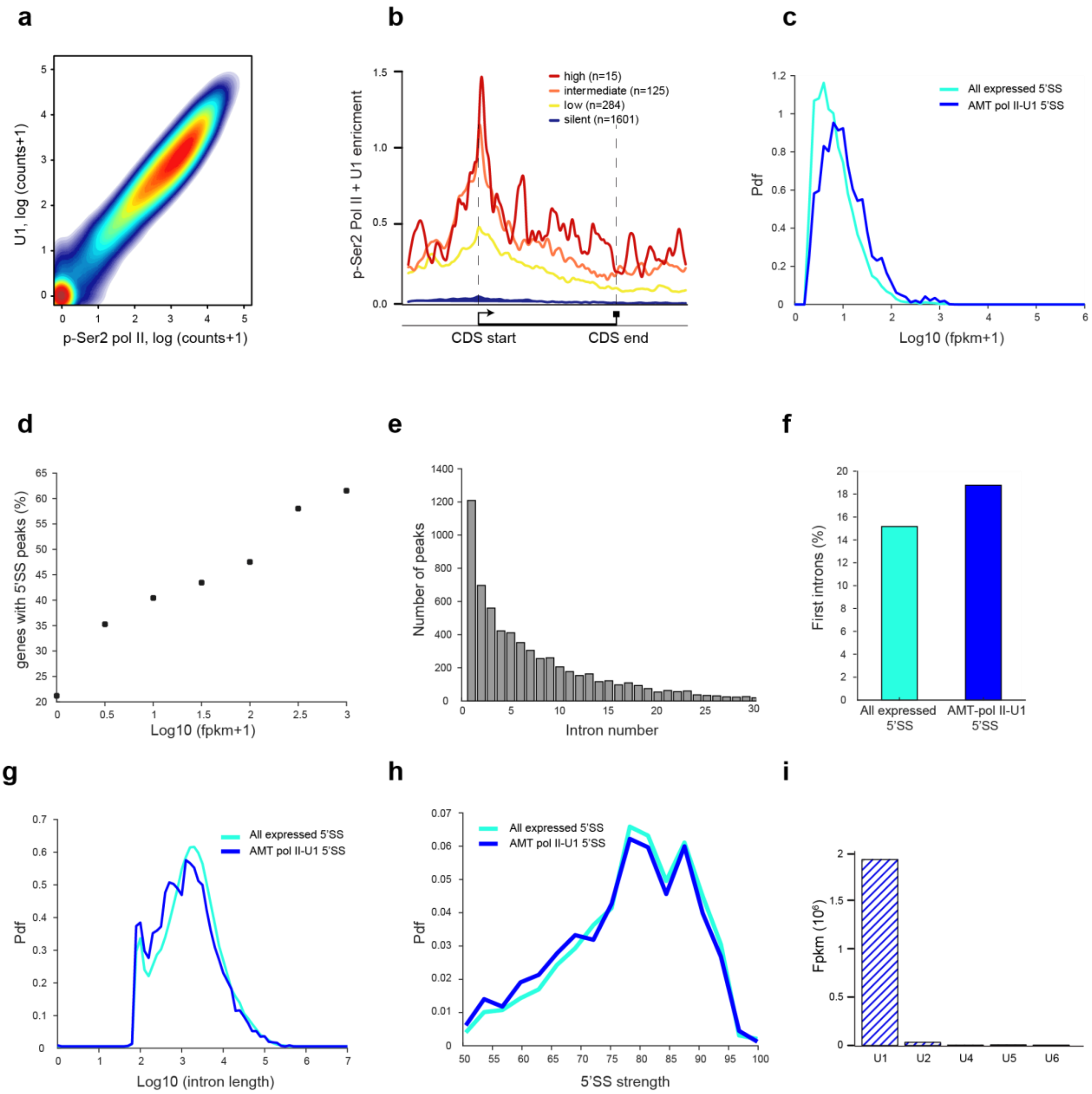

Supplementary Figure 5. p-Ser2 pol II and U1 travel together on expressed genes. a. p-Ser2 pol II and U1 ChIP signals are highly correlated. Pearson coefficient = 0.9887, $P = 2.2 \times 10^{-16}$, one-tailed correlation test. **b.** p-Ser2 pol II:U1C occupancy over intronless genes surrounded by 500 bp flanking sequences around the coding regions. Genes were divided based on expression (fpkm) into high, intermediate, low, and silent. Coding regions were scaled to 1000 bp. **c.** Expression distribution of pol II-U1-5'SS peaks in expressed genes compared to all 5'SS in expressed genes. **d.** Percentage of genes with pol II-U1-5'SS peaks among expressed genes

with different expression level thresholds. **e.** Intron number distribution of the pol II-U1-5’SS peaks in expressed genes. **f.** The fraction of first introns among pol II-U1-5'SS peaks in expressed genes compared to all 5'SS in expressed genes. One-tailed population proportion test z is -7.8407, $P < 10^{-10}$. N=126,770 expressed junctions. **g.** Intron length and **h.** 5’SS strength of pol II-U1-5’SS peaks in expressed genes compared to all 5'SS in expressed genes. **i.** Spliceosomal snRNA levels (fpkm) in double RNA-ChIP sample, Pdf – probability density function.

Supplementary Tables

Supplementary Table 1: Oligonucleotide sequences used in the cloning of single guide RNAs. Each sgRNA oligonucleotide was annealed to its complementary oligonucleotide before cloned into pX552 sgRNA expression plasmid. All sgRNAs used in this work are listed.

Name	Sequence (5' - 3')	Notes
FRG1- intron 1-1	ATCCCAGGAGATTCTCGGTA	Targets intron 1 of *FRG1* minigene
FRG1- intron 1-2	TTACCTTACCGAGAATCTCC	Targets intron 1 of *FRG1* minigene
FRG1- intron 2-1	GGCACAGGTTTAAATCGTCT	Targets intron 2 of *FRG1* minigene
FRG1- intron 2-2	AGTATCTCATCCTAACTAGT	Targets intron 2 of *FRG1* minigene
PLOD1-1	GTTCCCGGAAACAATTACC	Targets intron 17 of *PLOD1* gene
PLOD1-2	GAATTGTGAGAATTAACCG	Targets intron 17 of *PLOD1* gene
YBX1-1	AACCCTTAAGGGTTGCCAAC	Targets intron 3 of *YBX1* gene
YBX1-2	CTGCTATCCCCTATGACTGC	Targets intron 3 of *YBX1* gene
AKIRIN2-1	ATGTTTCAACCACTAATACC	Targets intron 2 of *AKIRIN2* gene
AKIRIN2-2	GGGCACTAGACTGTATTAGC	Targets intron 2 of *AKIRIN2* gene
hnRNPC-1	GCCTTAGACAGTTCCTAATT	Targets intron 2 of *hnRNPC* gene
hnRNPC-2	TAGAGTTCTAATGCTAGATT	Targets intron 2 of *hnRNPC* gene
PREP-1	TCACCGGTTCAACCACTAAG	Targets intron 9 of *PREP* gene
PREP-2	GCGGCAAACTTTGTAACCCA	Targets intron 9 of *PREP* gene

Supplementary Table 2: Oligonucleotide sequences used in PCR and quantitative PCR. Primer sequences are included for different assays: splicing by RT-PCR of *FRG1* minigene and endogenues genes, qRT-PCR for co-transcriptional analysis, ChIP and RNA ChIP experiments.

Name	Sequence (5' - 3')	Assay
FRG1 minigene-F	AAAGGTACCTTGATGAGGGCCCTAGTCCTCC	Used in *FRG1* minigene construction
FRG1 minigene-R	AAAGGATCCCTTGATCATTTCTTCTTCTCC	Used in *FRG1* minigene construction
U1-F-1	CCATGATCACGAAGGTGGTTT	Used in RNA ChIP-qPCR to amplify U1snRNA
U1-R-1	ATGCAGTCGAGTTTCCCACAT	Used in RNA ChIP-qPCR to amplify U1snRNA
U2-F	TCCCAGGGCGAGGCTTATCCATT	Used in RNA ChIP-qPCR to amplify U2snRNA
U2-R	GAACGCAGTCCCCCACTACCACAAAT	Used in RNA ChIP-qPCR to amplify U2snRNA
U4-F	CTCGGCCTTTTGGCTAAGAT	Used in RNA ChIP-qPCR to amplify U4snRNA
U4-R	TATTCCATCTCCCTGCTCCA	Used in RNA ChIP-qPCR to amplify U4snRNA
U5-F	GATCATTTCTATAGTGTGTTACTAGA	Used in RNA ChIP-qPCR to amplify U5snRNA
U5-R	CAATACGGAGAGAAGAACGATC	Used in RNA ChIP-qPCR to amplify U5snRNA
U6-F	GCAGTATCGTAGCCAATGAGG	Used in RNA ChIP-qPCR to amplify U6snRNA
U6-R	CTGTCAAAAATTGCCAGTGCC	Used in RNA ChIP-qPCR to amplify U6snRNA

FRT Fw	GTTTTGACCTCCATAGAAGACAC	Used in PCR to check *FRG1* splicing and in qPCR to amplify spliced transcript (“spliced 1”)
FRT Rv	CTGATCAGCGGGTTTAAACGT	Used in PCR to check *FRG1* splicing and in qPCR to amplify spliced transcript (“spliced 2”)
R-juncEX2_EX1-FRG1	TCA GGG CGA TTC TGG AAT CAG	Used in qPCR to amplify spliced co-transcriptional *FRG1* transcripts (“spliced 1”)
F-uniqex1	TAGTCCTCCAGAGCAGTTTAC	Used in qPCR to amplify unspliced co-transcriptional *FRG1* transcripts (“spliced 1”)
FRG-in1-R	CTGTGGGCTTAAGACAGATAC	Used in qPCR to amplify unspliced co-transcriptional *FRG1* transcripts (“spliced 1”)
F-juncEX2-EX3-FRG1	GTCTTTCAAAATGGGAAAATGGC	Used in qPCR to amplify spliced co-transcriptional *FRG1* transcripts (“spliced 2”)
FRG1_ex2 splicing_F	GAACAATGGGAACCAGTCTTTC	Used in qPCR to amplify unspliced co-transcriptional *FRG1* transcripts (“spliced 2”)
FRG-in2-R	TATGATTGACTGTTAACAGACTTTCT	Used in qPCR to amplify unspliced co-transcriptional *FRG1* transcripts (“spliced 2”)
F-uniq in1 BS	ACCACTCAGCCACATGCAG	Used in qPCR to amplify brance site sequence of *FRG1* intron 1. Used for U2 and U2AF65 RNA ChIP experiments.
R-uniq in1 BS	GGACATCAAAACAGGACATGC	Used in qPCR to amplify brance site sequence of *FRG1* intron 1. Used for U2 and U2AF65 RNA ChIP experiments.

FRG1-in2gRNAcheck-F	CCAGCACACATGTATAACTGAAG	Used in PCR for checking sgRNA efficient.
in2gRNAcheck-R	GCTTAACTCCCTTAACAAATATAATG	Used in PCR for checking sgRNA efficient.
FRT_Fw	GTTTTGACCTCCATAGAAGACAC	Used in qPCR for transcription inhibition by dCas9 in *FRG1* minigene experiment ("Upstream").
FRG-EX1-R	ACTGCTCTGGAGGACTAGG	Used in qPCR for transcription inhibition by dCas9 in *FRG1* minigene experiment ("Upstream").
FRG-in2near 3ss-F	CACAAGAAGTATCCTCATGGC	Used in qPCR for transcription inhibition by dCas9 in *FRG1* minigene experiment ("Downstream").
FRT_Rv	CTGATCAGCGGGTTTAAACGT	Used in qPCR for transcription inhibition by dCas9 in *FRG1* minigene experiment ("Downstream").
F-uniqex1	TAGTCCTCCAGAGCAGTTTAC	Used in qPCR for CRISPR interference protocol to amplify *FRG1* minigene regions (first 5'SS).
FRG-in1-R	CTGTGGGCTTAAGACAGATAC	Used in qPCR for CRISPR interference protocol to amplify *FRG1* minigene regions (first 5'SS).
F-uniqin1upsg	CAGAGAATGTTACAGAATCATAACG	Used in qPCR for CRISPR interference protocol to amplify *FRG1* minigene regions (negative control 1 intron 1 for sgRNA intron 2 or positive control for sgRNA intron 1).
R-uniqin1upsg	CTATACTTTTGCACAAGGTGAGA	Used in qPCR for CRISPR interference protocol to amplify *FRG1* minigene regions (negative control 1 intron 1 for sgRNA intron 2 or positive control for sgRNA intron 1).

FRG1-mid INTRON1-F	TGCGAACAATCAGATCTCAATAGA	Used in qPCR for CRISPR interference protocol to amplify *FRG1* minigene regions (negative control 2 intron 1).
R-FRG1-mid INTRON1	AGTATCCTATAAATTTGCATTTTAGTAGT	Used in qPCR for CRISPR interference protocol to amplify *FRG1* minigene regions (negative control 2 intron 1).
FRG1-JUNCTION-WT-F	CAATGGGAACCAGTCTTTCAAAATG	Used in qPCR for CRISPR interference protocol to amplify *FRG1* minigene regions (second 5’SS WT).
FRG1-JUNCTION-MUT-F	CAATGGGAACCAGTCTTTCAAAATA	Used in qPCR for CRISPR interference protocol to amplify *FRG1* minigene regions (second 5’SS MUT).
FRG-in2-R	TATGATTGACTGTTAACAGACTTTCT	Used in qPCR for CRISPR interference protocol to amplify *FRG1* minigene regions (second 5’SS).
FRG1-middle intron2-F	CAGTAAGGACCAATGGGCAC	Used in qPCR for CRISPR interference protocol to amplify *FRG1* minigene regions (negative control 3 intron 2).
R-FRG1-middle intron2	CTTAGAAAACACTGCTGAGTTCC	Used in qPCR for CRISPR interference protocol to amplify *FRG1* minigene regions (negative control 3 intron 2).
F-FRGIN2-near-sg	GACTTAGATAGTACTAATAATACAACA	Used in qPCR for CRISPR interference protocol to amplify *FRG1* minigene regions (positive control for sgRNA intron 2).
R-FRGIN2-near-sg	GCAATAGTTTTAAGACCCTCTTTC	Used in qPCR for CRISPR interference protocol to amplify *FRG1* minigene regions (positive control for sgRNA intron 2).
PLOD1_5ss_up	GAGGAGATGGAGCACTTTGG	Used in PCR to check *PLOD1* splicing
PLOD1_splice_R	GAGGGCTGCTCATCAGGC	Used in PCR to check *PLOD1* splicing
YBX1-ex2-F	CAACGAAGGTTTTGGGAACAG	Used in PCR to check *YBX1* splicing

YBX1_splice_R	CACAGTCTCTCCATCTCCTAC	Used in PCR to check *YBX1* splicing
F_PREPex7	TACAGCAGGAATCCAGTGGC	Used in PCR to check *PREP* splicing
R_PREPex10	TGGAATCTTCGTACCATCCTTG	Used in PCR to check *PREP* splicing
AKIRIN2_ex1_F	GTATCTCCGAATGGAGCCATC	Used in PCR to check *AKIRIN2* splicing
AKIRIN2_ex4_R	CATTATTTGATCATGCGTAAACTTCA	Used in PCR to check *AKIRIN2* splicing
HNRNPC_ex1_F	ACGAAGACTGAGCGGTTGTG	Used in PCR to check *hnRNPC* splicing
HNRNPC_ex4_R	CGCTGCAGATCGTTTCACAC	Used in PCR to check *hnRNPC* splicing
PLOD1_5ss_up	GAGGAGATGGAGCACTTTGG	Used in qPCR for CRISPR interference protocol to amplify *PLOD1* regions (first 5’SS).
PLOD1_5ss_up-R	AGAAGACCCCAGACAGTGAG	Used in qPCR for CRISPR interference protocol to amplify *PLOD1* regions (first 5’SS).
PLOD1_neg.cont1-F	CACTGTACTTAAAGGCAACATTAC	Used in qPCR for CRISPR interference protocol to amplify *PLOD1* regions (negative control 1 intron 1).
PLOD1_neg.cont1-R	GAGGTGGGTTGGGGCTTG	Used in qPCR for CRISPR interference protocol to amplify *PLOD1* regions (negative control 1 intron 1).
PLOD1_5ss _down-F	CAAATTCCTGCTGGAGTACATTG	Used in qPCR for CRISPR interference protocol to amplify *PLOD1* regions (second 5’SS).
PLOD1_5ss_down-R	CCCAGCCACTCCACTGAC	Used in qPCR for CRISPR interference protocol to amplify *PLOD1* regions (second 5’SS).

PLOD1_neg.cont3-F	AGTACGGGTGGAGGAACATC	Used in qPCR for CRISPR interference protocol to amplify *PLOD1* regions (negative control 2 intron 2).
PLOD1_neg.cont3-R	CACACGCTACTAGATGGAGG	Used in qPCR for CRISPR interference protocol to amplify *PLOD1* regions (negative control 2 intron 2).
PLOD1_pos.cont-F	GCCCTGCTTCGTCCGAAG	Used in qPCR for CRISPR interference protocol to amplify *PLOD1* regions (positive control).
PLOD1_pos.cont-R	GCATTCAAGGGATGAATGACAAC	Used in qPCR for CRISPR interference protocol to amplify *PLOD1* regions (positive control).
YBX1_5ss_up-F	GGAACGGATATGGTTTCATCAAC	Used in qPCR for CRISPR interference protocol to amplify *YBX1* regions (first 5’SS).
YBX1_5ss_up-R	CGCCTTTCAGAACAGAGCTG	Used in qPCR for CRISPR interference protocol to amplify *YBX1* regions (first 5’SS).
YBX1_neg.cont1-F	GTGTCAGACTGTGCAGGTG	Used in qPCR for CRISPR interference protocol to amplify *YBX1* regions (negative control 1 intron 1).
YBX1_neg.cont1-R	CAAGCTGTCAAGTTCCTATGG	Used in qPCR for CRISPR interference protocol to amplify *YBX1* regions (negative control 1 intron 1).
YBX1_5ss_down-F	CCAAGGAAGATGTATTTGTACAC	Used in qPCR for CRISPR interference protocol to amplify *YBX1* regions (second 5’SS).
YBX1_5ss_down-R	GCTGAGAACATATCTAACCTTTTC	Used in qPCR for CRISPR interference protocol to amplify *YBX1* regions (second 5’SS).
YBX1_neg.cont2-F	CTGATGAGTACTGCATAGTGAAC	Used in qPCR for CRISPR interference protocol to amplify *YBX1* regions (negative control 2 intron 2).

YBX1_neg.cont2-R	CACTTGGCAGTGGTTTACATTAG	Used in qPCR for CRISPR interference protocol to amplify *YBX1* regions (negative control 2 intron 2).
YBX1 pos.cnt-2 F	GTATTTGATGACAAGGAGGAAGC	Used in qPCR for CRISPR interference protocol to amplify *YBX1* regions (positive control).
YBX1 pos.cnt-2 R	AACATTTTGTCTGAAGTAGTCCTG	Used in qPCR for CRISPR interference protocol to amplify *YBX1* regions (positive control).
F_junc1_PREP	GTACTTGTTCCTGAGCATGAG	Used in qPCR for CRISPR interference protocol to amplify *PREP* regions (first 5’SS).
R_junc1_PREP	ACTAGAGAATGAGAGACGCAC	Used in qPCR for CRISPR interference protocol to amplify *PREP* regions (first 5’SS).
F-mid1 new2_PREP	TCTTCCTGCACCTCATCCAG	Used in qPCR for CRISPR interference protocol to amplify *PREP* regions (negative control 1 intron 1).
R-mid1 new2_PREP	GACTGGTGGGTTGAGGATC	Used in qPCR for CRISPR interference protocol to amplify *PREP* regions (negative control 1 intron 1).
F_junc2_PREP	AAGGACACTGAAATCTTCTATCAG	Used in qPCR for CRISPR interference protocol to amplify *PREP* regions (second 5’SS).
R_junc2_PREP	CATAGCATTATACTTCCATTTCCC	Used in qPCR for CRISPR interference protocol to amplify *PREP* regions (second 5’SS).
F_mid2_PREP	AACCTGGAGTACAGCAGCAG	Used in qPCR for CRISPR interference protocol to amplify *PREP* regions (negative control 2 intron 2).
R_mid2_PREP	ACTTCTGTACCCAGTGTAAATTTG	Used in qPCR for CRISPR interference protocol to amplify *PREP* regions (negative control 2 intron 2).

F_nSG_PREP	GACATTTGATAACTCTGGCAGG	Used in qPCR for CRISPR interference protocol to amplify *PREP* regions (positive control).
R_nSG_PREP	GTAAGTCATAAATGGCTTGGGC	Used in qPCR for CRISPR interference protocol to amplify *PREP* regions (positive control).
F junc1 Akir	CTCACCACAGGTGGGACC	Used in qPCR for CRISPR interference protocol to amplify *AKIRIN2* regions (first 5’SS).
R junc1 Akir	TGCTAAGAACAAGAACCACACAGTC	Used in qPCR for CRISPR interference protocol to amplify *AKIRIN2* regions (first 5’SS).
AKIRIN2_int1F	TGCCCAGTTGTGAGTATCTTTC	Used in qPCR for CRISPR interference protocol to amplify *AKIRIN2* regions (negative control 1 intron 1).
AKIRIN2_int1R	CAACTATTCAAGAGGCCCAAAC	Used in qPCR for CRISPR interference protocol to amplify *AKIRIN2* regions (negative control 1 intron 1).
AKIRIN2_ex2int2F	ACCAGCTTCACCAGGTAATAAC	Used in qPCR for CRISPR interference protocol to amplify *AKIRIN2* regions (second 5’SS).
AKIRIN2_ex2int2R	GAGGAGATACAGTAGCTTAAATGAC	Used in qPCR for CRISPR interference protocol to amplify *AKIRIN2* regions (second 5’SS).
AKIRIN2_int2F3	GTACCTTGTCATGACTGAACAC	Used in qPCR for CRISPR interference protocol to amplify *AKIRIN2* regions (negative control 2 intron 2).
AKIRIN2_int2R3	ATAAGACAAGTGTGTGAGGCAG	Used in qPCR for CRISPR interference protocol to amplify *AKIRIN2* regions (negative control 2 intron 2).
AKIRIN2int2_seq_F	AAATCAAAGACCATAACATAGTC	Used in qPCR for CRISPR interference protocol to amplify *AKIRIN2* regions (positive control).

R near guide Akir	CAATGGGCATGGCAGTATTTAG	Used in qPCR for CRISPR interference protocol to amplify *AKIRIN2* regions (positive control).
Fjunc1_HNRNPC	CCCCTTCTTGGTAAGTGGAG	Used in qPCR for CRISPR interference protocol to amplify *hnRNPC* regions (first 5'SS).
HNRNPC_int1R	ACAAGCAGAATAAATGAGGGCC	Used in qPCR for CRISPR interference protocol to amplify *hnRNPC* regions (first 5'SS).
HNRNPC_int1F	GAATGGCTGTGCGAAACAAGC	Used in qPCR for CRISPR interference protocol to amplify *hnRNPC* regions (negative control 1 intron 1).
HNRNPC_int1R2	AACTCCTAGGCAAACTTACTCC	Used in qPCR for CRISPR interference protocol to amplify *hnRNPC* regions (negative control 1 intron 1).
HNRNPC_ex2int2F	TTTTGATCTTCAGCTACAGTAAG	Used in qPCR for CRISPR interference protocol to amplify *hnRNPC* regions (second 5'SS).
HNRNPC_int2R4	GTGTCATTATCATCATCATCATC	Used in qPCR for CRISPR interference protocol to amplify *hnRNPC* regions (second 5'SS).
HNRNPC_int2F3	CTGAAAGCCATCTTCCTCCTG	Used in qPCR for CRISPR interference protocol to amplify *hnRNPC* regions (negative control 2 intron 2).
HNRNPC_int2R3	CTGGGCATCCATGTTGCTG	Used in qPCR for CRISPR interference protocol to amplify *hnRNPC* regions (negative control 2 intron 2).
F_nSG3_HNRNPC	AAGAGTCAAACCATAGGAGATAC	Used in qPCR for CRISPR interference protocol to amplify *hnRNPC* regions (positive control).
R_nSG3_HNRNPC	ATTTCCCTGCTTTGTGAGGTC	Used in qPCR for CRISPR interference protocol to amplify *hnRNPC* regions (positive control).

FRG_mutx2_R	GAAGTTTTTATAAACAATAACAGCTCTC	Used in qPCR for CRISPR interference protocol to amplify *FRG1* minigene regions (second 5'SS MUTx2).
