# Supplementary material for: The upstream 5′ splice site remains associated to the transcription machinery during intron synthesis
Source: Nat Commun. 2021 Jul 27;12:4545. doi: 10.1038/s41467-021-24774-6 (PMC8316553; doi:10.1038/s41467-021-24774-6)
Supplement: Supplementary file 2 — Reporting Summary [file 41467_2021_24774_MOESM2_ESM.pdf]

nature research

Corresponding author(s): Gil Ast, Galit Lev Maor

Last updated by author(s): Jun 15, 2021

Reporting Summary

Nature Research wishes to improve the reproducibility of the work that we publish. This form provides structure for consistency and transparency in reporting. For further information on Nature Research policies, see our Editorial Policies and the Editorial Policy Checklist.

Statistics

For all statistical analyses, confirm that the following items are present in the figure legend, table legend, main text, or Methods section.

n/a	Confirmed	
☐	☒	The exact sample size (n) for each experimental group/condition, given as a discrete number and unit of measurement
☐	☒	A statement on whether measurements were taken from distinct samples or whether the same sample was measured repeatedly
☐	☒	The statistical test(s) used AND whether they are one- or two-sided<br>*Only common tests should be described solely by name; describe more complex techniques in the Methods section.*
☐	☒	A description of all covariates tested
☐	☒	A description of any assumptions or corrections, such as tests of normality and adjustment for multiple comparisons
☐	☒	A full description of the statistical parameters including central tendency (e.g. means) or other basic estimates (e.g. regression coefficient) AND variation (e.g. standard deviation) or associated estimates of uncertainty (e.g. confidence intervals)
☐	☒	For null hypothesis testing, the test statistic (e.g. F, t, r) with confidence intervals, effect sizes, degrees of freedom and P value noted<br>*Give P values as exact values whenever suitable.*
☒	☐	For Bayesian analysis, information on the choice of priors and Markov chain Monte Carlo settings
☒	☐	For hierarchical and complex designs, identification of the appropriate level for tests and full reporting of outcomes
☐	☒	Estimates of effect sizes (e.g. Cohen's d, Pearson's r), indicating how they were calculated

Our web collection on statistics for biologists contains articles on many of the points above.

Software and code

Policy information about availability of computer code

Data collection	No software was used
Data analysis	trimmomatic software version 0.39<br>Bowtie2 v2.1.0<br>Bowtie2 2.4.2<br>STAR aligner v2.7.1a<br>bam2wig.pl (http://search/cpan.org/~rjparnell/Bio-ToolBox-1.44)<br>bwtool v1.0<br>Cufflinks v2.2.1<br>MaxEntScan::score5ss<br>R package bamsignals

For manuscripts utilizing custom algorithms or software that are central to the research but not yet described in published literature, software must be made available to editors and reviewers. We strongly encourage code deposition in a community repository (e.g. GitHub). See the Nature Research guidelines for submitting code & software for further information.

Data

Policy information about availability of data

All manuscripts must include a data availability statement. This statement should provide the following information, where applicable:

- Accession codes, unique identifiers, or web links for publicly available datasets
- A list of figures that have associated raw data
- A description of any restrictions on data availability

All ChIP-seq, RNA ChIP-seq, and RNA-seq raw and processed data generated in this study have been deposited in and are publically available in the GEO database under accession code GSE145092 (https://www.ncbi.nlm.nih.gov/geo/query/acc.cgi?acc=GSE145092). ..Source data underlying Figs......... are provided as a Sourc.e data file.

Field-specific reporting

Please select the one below that is the best fit for your research. If you are not sure, read the appropriate sections before making your selection.

- [x] Life sciences
- [ ] Behavioural & social sciences
- [ ] Ecological, evolutionary & environmental sciences

For a reference copy of the document with all sections, see nature.com/documents/nr-reporting-summary-flat.pdf

Life sciences study design

All studies must disclose on these points even when the disclosure is negative.

Sample size	RNA-seq (Fig 4 and Sup 5)- 1 replicate, ChIP-seq (Fig 4 and Sup 5)- 1 replicate, RNA ChIP-seq (Fig 4 and Sup 5)- duplicate, CRISPR RNA ChIP-seq (Fig 3d)- 1 replicate, RNA ChIP (Sup 2a)-1 replicate Fig 1b-1 replicate. All other experiments were performed in triplicate to access statistical significance.
Data exclusions	No data was excluded from our analyses.
Replication	RNA-seq (Fig 4 and Sup 5)- 1 replicate, ChIP-seq (Fig 4 and Sup 5)- 1 replicate, RNA ChIP-seq (Fig 4 and Sup 5)- duplicate, CRISPR RNA ChIP-seq (Fig 3d)- 1 replicate, RNA ChIP (Sup 2a)-1 replicate Fig 1b-1 replicate. All other experiments were performed in triplicate to access statistical significance. All replicate experiments were concordant.
Randomization	No live organisms/preticipants/samples from patients were used in this study, only cell lines. cells were plated and assigned randomly as control or treatment samples at the beginning of each experiment.
Blinding	No blinding was required in our study as no human or animal subjects were used.

Reporting for specific materials, systems and methods

We require information from authors about some types of materials, experimental systems and methods used in many studies. Here, indicate whether each material, system or method listed is relevant to your study. If you are not sure if a list item applies to your research, read the appropriate section before selecting a response.

Materials & experimental systems

n/a	Involved in the study	
[ ]	[x]	Antibodies
[ ]	[x]	Eukaryotic cell lines
[x]	[ ]	Palaeontology and archaeology
[x]	[ ]	Animals and other organisms
[x]	[ ]	Human research participants
[x]	[ ]	Clinical data
[x]	[ ]	Dual use research of concern

Methods

n/a	Involved in the study	
[ ]	[x]	ChIP-seq
[x]	[ ]	Flow cytometry
[x]	[ ]	MRI-based neuroimaging

Antibodies

Antibodies used	anti-pol II p-Ser2 (Abcam; ab5095) (1:500), anti-pol II p-Ser5 (Abcam; ab5408) (1:1000), anti SNRPC (U1C) (Abcam; ab157116) (1:200), anti-U2AF2 (a gift of Prof. Juan Valcarcel, Centre for Genomic Regulation, Barcelona, Spain) (1:500), anti-U2AF35 (Abcam; ab172614) (1:250), anti-FUS (Abcam; ab23439) (1:400), anti-SAP155/SF3B1 (MBL; D221-3) (1:1000), anti-NXF1/TAP (Santa Cruz Biotechnology; sc- 32319) (1:500), anti-GAPDH (GenScript; A00191-40) (1:1000), anti-PTBP1 (Abcam; ab133734) (1:5000), anti a-tubulin (abcam; ab18251) (1:40000), anti-histone 4 (Millipore; 05-858) (1:30000), donkey anti-rabbit IgG (Abcam; ab97064) and goat anti-mouse IgG (Abcam; ab7068).

Validation	anti-pol II p-Ser2 (Abcam; ab5095)- Fu C et al. BRF Negatively Regulates Thermotolerance Defect of fes1a in Arabidopsis. Front Plant Sci 11:171 (2020).<br>anti-pol II p-Ser5 (Abcam; ab5408)- Bou Sleiman M et al. Enteric infection induces Lark-mediated intron retention at the 5' end of Drosophila genes. Genome Biol 21:4 (2020).<br>anti SNRPC (U1C) (Abcam; ab 157116) (1:200), no ref for that antibody<br>anti-U2AF2 (a gift of Prof. Juan Valcarcel, Centre for Genomic Regulation, Barcelona, Spain) (1:500), U2AF65 antibody was described in that article:https://www.sciencedirect.com/science/article/pii/S1097276512000329.<br>anti-U2AF35 (Abcam; ab 172614) (1:250) ab172614 has been referenced in 2 publications.<br>Esfahani MS et al. Functional significance of U2AF1 S34F mutations in lung adenocarcinomas. Nat Commun 10:5712 (2019).PubMed: 31836708<br>Palangat M et al. The splicing factor U2AF1 contributes to cancer progression through a noncanonical role in translation regulation. Genes Dev 33:482-497 (2019).PubMed: 30842218<br>anti-FUS (Abcam; ab23439) (1:400), according to datasheet cited 22 times<br>anti-SAP155/SF3B1 (MBL; D221-3) (1:1000), according to datasheet cited 11 times<br>anti-NXF1/TAP (Santa Cruz Biotechnology; sc- 32319) (1:500), according to datasheet selected cited 7 times<br>anti-GAPDH (GenScript; A00191-40) (1:1000), according to datasheet selected cited 10 times<br>anti-PTBP1 (Abcam; ab133734) (1:5000), according to datasheet cited 7 times<br>anti a-tubulin (abcam; ab 18251) (1:40000), according to datasheet cited 234 times<br>anti-histone 4 (Millipore; 05-858) (1:30000), according to datasheet cited 58 times<br>donkey anti-rabbit IgG (Abcam; ab 97064) according to datasheet cited 24 times<br>and goat anti-mouse IgG (Abcam; ab 7068) according to datasheet cited 12 times

Eukaryotic cell lines

Policy information about cell lines

Cell line source(s)	Flp-In-HEK293 (Invitrogen), HEK293 (ATCC), and HeLa (ATCC)
Authentication	Cell lines were not authenticated
Mycoplasma contamination	Cell lines were tested negative for mycoplasma
Commonly misidentified lines<br>(See ICLAC register)	No commonly misidentified cell lines were used in this study

ChIP-seq

Data deposition

- [x] Confirm that both raw and final processed data have been deposited in a public database such as GEO.
- [x] Confirm that you have deposited or provided access to graph files (e.g. BED files) for the called peaks.

Data access links<br>*May remain private before publication.*	The datasets generated and analyzed during the current study are available in the GEO repository, GSE145092
Files in database submission	DNA-ChIP:<br>pol2_ser2.fastq.gz<br>U1.fastq.gz<br>pol2_ser2_U1.fastq.gz<br>input.fastq.gz<br>pol2_ser2_PTPBP1.fastq.gz<br>pol2_ser2_U2AF65.fastq.gz<br>U2AF65.fastq.gz<br><br>pol2_ser2.bw<br>U1.bw<br>pol2_ser2_U1.bw<br>input.bw<br>pol2_ser2_PTPBP1.bw<br>pol2_ser2_U2AF65.bw<br>U2AF65.bw<br>pol2_ser2_normalized.bw<br>U1_normalized.bw<br>pol2_ser2_U1_normalized.bw<br>pol2_ser2_PTPBP1_normalized.bw<br>pol2_ser2_U2AF65_normalized.bw<br>U2AF65_normalized.bw<br><br>RNA-ChIP:

pol2_ser2_U1_noAMT.fastq.gz
pol2_ser2_U1_AMT.fastq.gz
pol2_ser2_AMT.fastq.gz
input_AMT.fastq.gz
input_noAMT.fastq.gz
pol2_ser2_U1_AMT_rep2_R1.fastq.gz
pol2_ser2_AMT_rep2_R1.fastq.gz
input_AMT_rep2_R1.fastq.gz
pol2_ser2_U1_AMT_rep2_R2.fastq.gz
pol2_ser2_AMT_rep2_R2.fastq.gz
input_AMT_rep2_R2.fastq.gz
U1_149_R1.fastq.gz
U1_149_R2.fastq.gz
input_149_R1.fastq.gz
input_149_R2.fastq.gz
pol2_ser2_U1_noAMT.bw
pol2_ser2_U1_AMT.bw
pol2_ser2_AMT.bw
input_AMT.bw
input_noAMT.bw
pol2_ser2_U1_noAMT_normalized.bw
pol2_ser2_U1_AMT_normalized.bw
pol2_ser2_AMT_normalized.bw
149_U1_ctrl_aligned_to_U1_Mut_and_WT.bam
149_U1_IP_aligned_to_U1_Mut_and_WT.bam

Genome browser session (e.g. UCSC)

https://genome.ucsc.edu/index.html (hg38)

Methodology

Replicates

Two replicates for RNA-ChIP AMT samples and one replicate for the rest of the samples

Sequencing depth

DNA-ChIP:
No. of reads:
input: 86,705,671;pol2: 96,597,994;U1:94,346,442;pol2-U1: 115,304,779
Uniquely no. of reads:
input: 72,871,782;pol2: 83,103,090;U1: 80,894,252;pol2-U1: 97,017,802
Length of reads:
50bp
single-end

RNA-ChIP:
No. of reads:
pol2_ser2_U1_noAMT: 223515808; pol2_ser2_U1_AMT: 38880282 ;pol2_ser2_AMT: 91368693; input_AMT: 76189608; input_noAMT: 135791769;
pol2_ser2_U1_AMT_rep2: 159097024; pol2_ser2_AMT_rep2: 138895578; input_AMT_rep2: 129131298;
U1_149: 30927748; control_149: 26135531;

Uniquely no. of reads
noAMT-input: 74866260;AMT-input : 11790788;AMT-pol2-u1: 3348234;AMT-pol2: 10314128;no-AMT-pol2-u1: 5817163
AMT-input-rep2:51127968; AMT-pol2-rep2:37466160; AMT-pol2-U1-rep2:62454420
U1_149 : 243486; input_149 : 29968
Length of reads:
50bp, single-end + 75bp, paired-end

Antibodies

1) https://www.abcam.com/rna-polymerase-ii-ctd-repeat-ysptsps-phospho-s2-antibody-chip-grade-ab5095.html
2) https://www.abcam.com/u1-c-antibody-ab157116.html;https://www.abcam.com/u2af35u2af1-antibody-epr12649bab172614.html

Peak calling parameters

Reads were mapped to the human reference genome hg38 using either STAR aligner using default parameters, or with Bowtie2 using default parameters with –very-sensitive mode. Only uniquely mapped reads were kept and duplicate reads were removed.
For RNA-ChIP exon-intron junctions, peaks were inferred using custom code in MATLAB (see Methods). Briefly, the criterion for peak calling was at least 1.5 fold-change difference between normalized number of reads in sample compared to input.

Data quality

QC was carried out using fastQC and visual inspection using IGV. 4647 and 5262 peaks were significant at FDR levels of 0.05 and 0.1, respectively. 502 peaks were significant with fold-change greater than 5 between AMT-pol2-U1 and input samples.

Software	Software Bowtie2 v2.1.0, bam2wig.pl (http://search/cpan.org/~rjparnell/Bio-ToolBox-1.44)<br>bwtool.
